# Effect of Surface Characteristics on Contact and Leakage in Sealed Joints with Metal Gaskets: A Review

**DOI:** 10.3390/ma19173658

**Published:** 2026-08-28

**Authors:** Anna Piwowar, Przemysław Jaszak

**Affiliations:** Faculty of Mechanical and Power Engineering, Wroclaw University of Science and Technology, Wybrzeze Wyspianskiego 27, 50-370 Wroclaw, Poland

**Keywords:** metal gasket, sealed joint, surface characteristics, contact, leakage

## Abstract

**Highlights:**

- Surface characteristics strongly affect the sealing performance of joints with metal gaskets.
- Reliable prediction of leakage at sealing interfaces requires coupled contact and microgap flow descriptions.
- Surface engineering and manufacturing-process optimization can improve sealing performance.

**Abstract:**

The article presents a review of the current state of knowledge on the effect of surface characteristics on contact conditions and leakage in sealed joints with metal gaskets. The sealing mechanism is discussed, in which joint tightness is governed primarily by the deformation of microscale surface irregularities, the increase in the real contact area, and the disruption of the continuity of microgaps forming flow channels. Particular attention is given to the influence of contact geometry and surface topography on the leakage rate. The paper summarizes selected experimental studies on metal gaskets and discusses approaches to modeling the contact of rough surfaces and leakage at their interface. Taken together, the reviewed evidence indicates that reliable prediction of tightness requires simultaneous consideration of contact mechanics, material properties, loading conditions, and the surface geometric structure. The review identifies promising directions for the further development of contact and leakage models. The importance of modeling and the deliberate design of sealing and mating surfaces for improving joint tightness and reducing fluid losses and fugitive emissions is also highlighted.

## 1. Introduction

Metal gaskets are used in joints operating under demanding service conditions, including reduced or elevated temperatures, high pressure, vacuum technology, chemically aggressive environments, and applications subject to particularly stringent tightness requirements or other requirements that are difficult to meet, such as high resistance to aging-related degradation [1], mechanical vibration [2], or gasket blowout [3], which would be impossible to achieve using non-metallic gaskets [4,5,6]. Their use is also justified in cases where long-term maintenance of contact pressure is of significant importance, especially under elevated temperature and loading conditions, in which creep or stress relaxation of the gasket material could lead to deterioration of tightness [7,8,9]. Metal gaskets are widely used, among others, in the aerospace, chemical, and nuclear power industries [10,11]. In such applications, joint tightness is critical for the safe operation of systems and for limiting fluid losses, whereas loss of tightness may lead to costly repairs and downtime and, in extreme cases, pose a threat to human life and the natural environment [12,13,14,15,16].

The importance of sealing performance is well illustrated by data from industrial installations, where thousands of gasketed joints are used, with flanged joints representing the largest group. According to the European Sealing Association (ESA) report [17], a typical oil refinery may contain at least 20,000 flanged joints. Losses from an individual gasketed joint are generally of limited magnitude; however, their cumulative effect may be significant: fugitive emissions from a single refinery in Europe may range from 600 to 10,000 tons of volatile organic compounds per year. In one of the European plants analyzed, equipment leaks accounted for approximately 72% of total volatile organic compound emissions, of which approximately 5% was attributed to flanged joints. These data show that gasket effectiveness in individual joints is important not only locally but, above all, globally, especially in large-scale industrial installations. Consequently, improving the sealing performance of individual joints through measures including appropriate control of surface characteristics may contribute to reducing cumulative emissions from industrial installations.

In recent years, increasing emphasis has been placed on reducing emissions and leakage from industrial and energy installations, as reflected in recently adopted European Union legislation. Commission Implementing Decision (EU) 2022/2427 [18] identifies flanges and other static connections as potential sources of fugitive emissions of volatile organic compounds and includes leak detection and repair programs among the best available techniques. Regulation (EU) 2024/1787 [19] establishes requirements for the measurement, monitoring, reporting, and reduction in methane emissions in the energy sector, whereas Directive (EU) 2024/1788 [20] addresses the development of hydrogen systems and requires measures to prevent and minimize hydrogen emissions, including regular leak detection and repair surveys. These legislative developments underscore the growing importance of reliable leakage assessment, prediction, and reduction.

In parallel with these regulatory changes, increasing attention has been paid to the effect of appropriate contact surface finishing on the operating conditions of joints, particularly through the formation of surface characteristics such as roughness, hardness, elastic-plastic properties, and contact stiffness [21,22,23]. With respect to sealed joints with metal gaskets, the contact conditions at the interface between the gasket and the mating surfaces are among the key factors affecting sealing performance. Due to microgeometric surface irregularities, real contact occurs only over limited areas; consequently, discontinuities may remain between the mating surfaces, promoting the formation of leakage paths [24,25]. Thus, joint tightness is determined not only by the load and material properties of the components, but also by the surface geometric structure [4,26,27].

For the purposes of this study, the surface geometric structure is considered at two scales: contact geometry and surface topography. Contact geometry refers to the macroscopic shape of the mating surfaces, such as flat, cylindrical, or spherical, and to the resulting nature of the contact, which may take the form of surface, line, or point contact. Surface topography, in contrast, denotes the microgeometric description of the surface, including roughness, waviness, and the directionality of machining marks.

Advances in precision manufacturing, digital areal metrology, and computational modeling have progressively expanded the possibilities for designing and analyzing sealing surfaces. Precision machining and controlled texturing enable the deliberate shaping of surface microgeometry [28], whereas digital areal measurements provide information on the three-dimensional arrangement and directionality of surface irregularities that is not fully captured by parameters based on two-dimensional profiles [29,30]. At the same time, the ongoing development of analytical, semi-analytical, and numerical models enables increasingly accurate modeling of rough-surface contact and leakage at the interface. Multiscale approaches are also being used increasingly often to couple global contact conditions determined by the geometry and loading of the entire joint with a microscale description of rough-surface contact and fluid flow through the resulting microgaps [31,32,33,34]. In recent years, the continued development and integration of these methods have provided more detailed measurement data and more advanced computational tools for predicting leakage based on the physical description of phenomena occurring at the sealing interface.

Despite numerous studies on the effect of the characteristics of sealing and mating surfaces on contact conditions and leakage at their interface, as well as on methods for modeling these phenomena in sealed joints with metal gaskets, these issues have not yet, to the authors’ knowledge, been comprehensively discussed in a single review paper. Recent studies increasingly incorporate advanced surface characterization and new approaches to contact and leakage modeling, providing a timely basis for a comprehensive review. Such a review is needed now not only to consolidate the growing body of experimental and modeling research, but also to assess the capabilities and limitations of current leakage prediction approaches in light of increasingly stringent requirements for controlling and reducing leakage and emissions. The aim of this article is to review and systematize the current state of knowledge on the effect of surface characteristics on contact and leakage in joints of this type.

The literature review was conducted based on studies retrieved from the Scopus and Web of Science databases, supplemented with sources identified through citation analysis of selected publications. The search covered studies on metal gaskets, surface characteristics, contact modeling, and leakage modeling. Combinations of keywords corresponding to the main thematic areas of the article were used, including: metal gasket, static seal, flanged joint, surface roughness, surface topography, surface texture, sealing surface, contact model, leakage, leakage modeling, leakage prediction, and fluid permeability. Publications concerning sealed joints with metal gaskets were selected for further analysis, with particular emphasis on studies addressing the effects of contact geometry and surface topography on sealing performance, rough-surface contact modeling, and leakage modeling.

The article is organized as follows. Section 2 presents the fundamentals of the sealing mechanism in sealed joints with metal gaskets. Section 3 reviews studies on the effects of contact geometry and surface topography on joint tightness. Section 4 provides a review of selected approaches to modeling rough-surface contact and leakage at the interface. Section 5 identifies promising directions for further research. The article concludes with a summary of the main findings resulting from the literature review.

## 2. Fundamentals of Sealing Mechanism in Sealed Joints with Metal Gaskets

Metal surfaces, even those that appear smooth, are always characterized by roughness resulting from microscale surface irregularities [24,35]. Consequently, the real contact between two metal surfaces occurs only in selected regions corresponding to the highest peaks of the irregularities. Microgaps occur between them and constitute potential leakage paths [24,27].

By definition, a static seal (gasket) operates under conditions in which there is no relative motion between the sealed surfaces. A special case is represented by gasketed joints subjected to forced mechanical vibrations induced by equipment that is an integral part of a piping system, such as pumps, fans, or blowers. The tightness of a sealed joint is achieved by applying an appropriate contact pressure, which causes deformation of the microscale surface irregularities of the gasket surface and/or the mating surface, thereby closing the microgaps present in the contact region [36,37]. The minimum contact pressure on the gasket surface, applied during joint assembly, should be selected so that, during operation of the installation, increases in pressure or temperature, as well as the action of external loads on the pipeline, including transverse and axial forces and bending moments, do not cause it to decrease below the value required to ensure the intended joint tightness [38,39].

**Figure 1:**
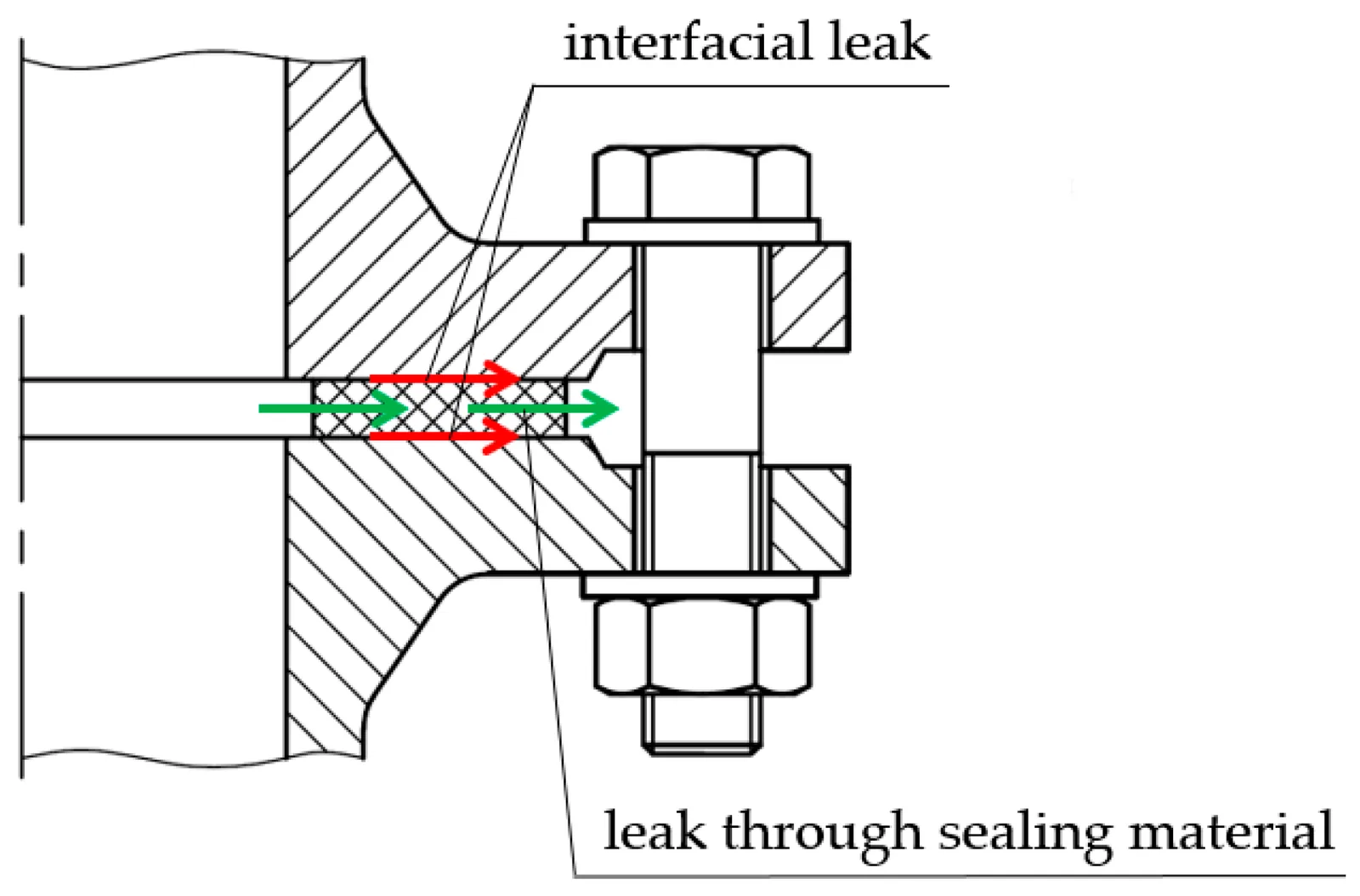
Components of leakage from a gasketed joint, based on [41].

The total leakage from a gasketed joint is the sum of leakage occurring at the surface interface and leakage through the gasket itself, as shown in Figure 1 [40,41]. In the case of joints with metal gaskets, leakage in the contact region between the sealing and mating surfaces is dominant, because dense metals exhibit extremely low through-material permeability, which is generally negligible under typical sealing conditions [40,42,43]. Consequently, through-gasket leakage, i.e., fluid transport through the material structure, is typically several to more than ten orders of magnitude lower than interfacial leakage. In contrast, the effective permeability of the interface formed by two rough surfaces strongly depends on the contact conditions. As the contact pressure increases, interacting surface asperities deform, reducing the dimensions of the remaining microgaps and, consequently, the permeability of the sealing interface. In practice, this means that fluid transport occurs primarily through microgaps between the irregularities of the sealing and mating surfaces, and that the leakage rate depends on the size of these microgaps [37].

**Figure 2:**
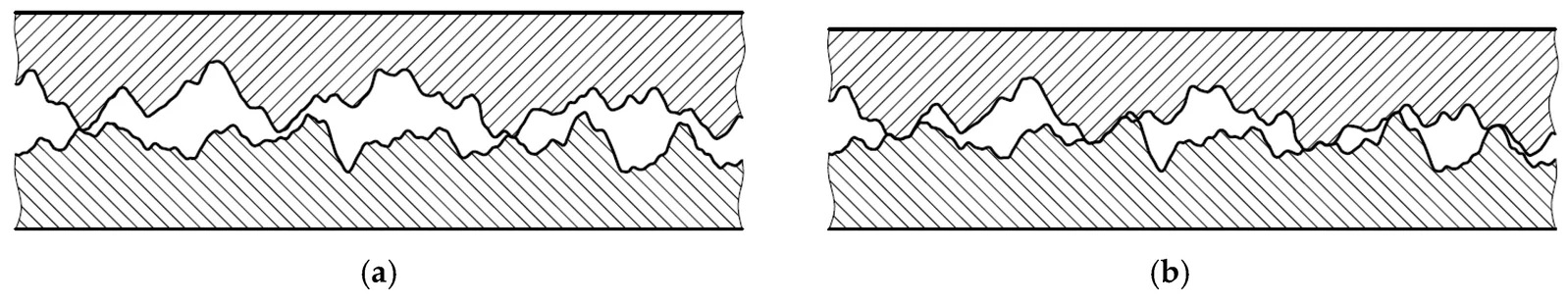
Stages of surface contact: (**a**) initial position; (**b**) state after load application, based on [48].

Surface contact conditions are affected by the magnitude of the applied load and the material properties of the mating components. In the initial state, contact between the surfaces occurs only in the regions of the highest asperities. As the load increases, microscale surface irregularities undergo partial deformation, and additional, lower asperities are progressively brought into contact, as shown in Figure 2 [44,45]. Deformation in the regions of microscale surface irregularities may be elastic, elastic-plastic, plastic, or plastic with material hardening [46,47]. As a result of this process, the original distribution, shape, and dimensions of the microscale surface irregularities change, while the real contact area gradually increases. At the same time, the dimensions of the microgaps decrease, and some potential flow channels are closed or interrupted, thereby limiting fluid transport [37]. Thus, the sealing mechanism in sealed joints with metal gaskets consists primarily in increasing the real contact area and limiting the continuity of microgaps forming leakage paths under the applied load.

Because the course of the phenomena described above depends largely on the characteristics of the sealing and mating surfaces, the sealing mechanisms described in this section provide the basis for the following analysis of how contact geometry and surface topography affect sealing performance in sealed joints with metal gaskets.

## 3. Influence of Surface Characteristics on Sealing Performance

The properties of sealing and mating surfaces in sealed joints with metal gaskets affect sealing performance both at the macroscopic scale, associated with contact geometry, and at smaller scales, including roughness, the directionality of machining marks, and related surface features [31,32,33]. These scales are interrelated because contact geometry determines the intensity of contact deformation between the mating surfaces, whereas surface topography defines the microgeometry of the gaps forming flow channels. The following subsections present a review of studies on the effects of contact geometry and surface topography on sealing performance in sealed joints with metal gaskets.

### 3.1. Influence of Interface Geometry on Sealing Performance

The contact geometry between a metal gasket and the mating surfaces is one of the fundamental factors determining sealing effectiveness. The contact pressure distribution, real contact area, and development of potential leakage paths depend not only on the axial load and the material properties of the components, but also on the surface contact geometry, defined by the dimensions and shape of the nominal contact zone. In the case of metal gaskets, this issue is particularly important because metals exhibit limited compliance, and achieving tightness requires sufficiently high contact pressures in the real contact regions. For this reason, the nominal contact area is often intentionally limited in metal gaskets. This results in the concentration of contact pressure within a smaller region, promoting deformation of surface irregularities and closure of leakage channels.

In practice, sealing performance is improved by appropriately shaping the contact geometry and selecting the gasket material [49]. The use of more compliant materials may promote better conformity of the gasket to the irregularities of the mating surfaces and increase the real contact area [50,51]. However, increasing the local contact pressure by reducing the nominal contact area involves a design trade-off. A narrow contact zone promotes the deformation of surface irregularities and microgap closure but, at high local contact pressures, may also lead to permanent local modification of the mating surface [52]. In contrast, a wider sealing zone may better tolerate local geometric surface defects [28], although under the same axial load it generally provides a lower mean contact pressure. Therefore, sealing effectiveness should not be evaluated solely in terms of contact pressure.

**Figure 3:**
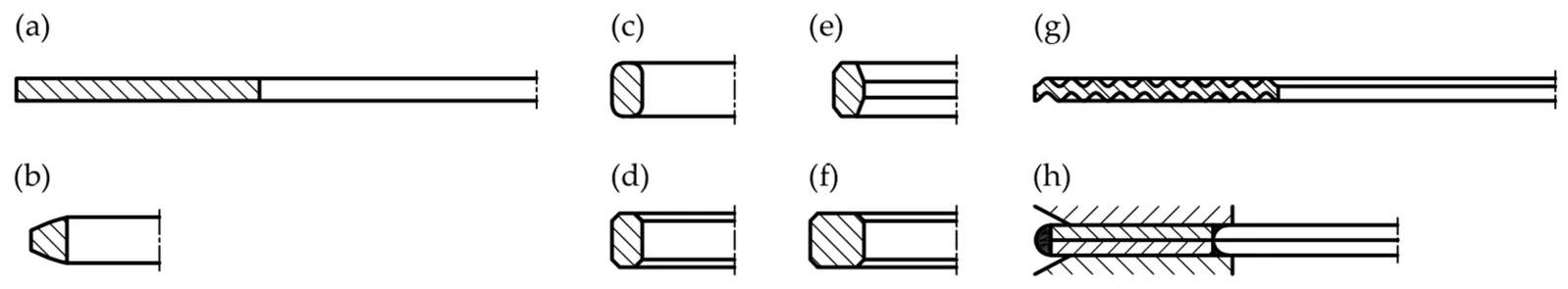
Basic types of metal gaskets: (**a**) flat gasket; (**b**) lenticular gasket; (**c**) RTJ oval gasket; (**d**) RTJ octagonal gasket; (**e**) RTJ RX gasket; (**f**) RTJ BX gasket; (**g**) corrugated gasket; (**h**) weld ring gasket.

The selection of a metal gasket design depends primarily on the required sealing performance, the magnitude of the applied load, fluid pressure, and joint geometry [22,53,54]. The basic designs include flat gaskets (Figure 3a), lenticular gaskets (Figure 3b), RTJ (Ring Type Joint) gaskets, including oval (Figure 3c), octagonal (Figure 3d), RX (Figure 3e), and BX (Figure 3f) types, corrugated gaskets (Figure 3g), and weld ring gaskets (Figure 3h) [36,55].

A flat gasket, i.e., one with a rectangular cross-section, has the advantage of a simple design because it can be cut from sheet metal of the required thickness. Its disadvantage is the nonuniform contact pressure distribution resulting from bolt tightening and flange bending. A lenticular gasket addresses these problems by adapting well to such deformations. Oval, octagonal, RX, and BX gaskets are used in very high-pressure applications. They are installed in specially designed flanges with grooves machined into the flange faces, resulting in a combined form-fit and frictional connection between the gasket and the flange. To increase and concentrate the contact pressure, a corrugated geometry is used. The corrugations on the gasket face produce local concentrations of contact pressure, thereby improving sealing performance at relatively low bolt preload. A disadvantage of these designs is that they may cause damage to the flange sealing surfaces as a result of plastic deformation. The membrane gasket, welded to the flanges, acts as a compensator for thermal and mechanical deformations. It provides the highest level of joint tightness and is used in installations with the highest safety requirements. Its disadvantage is the difficult installation and removal process, which involves welding and subsequent removal of the welds and therefore requires specialized equipment and qualified personnel.

**Figure 4:**
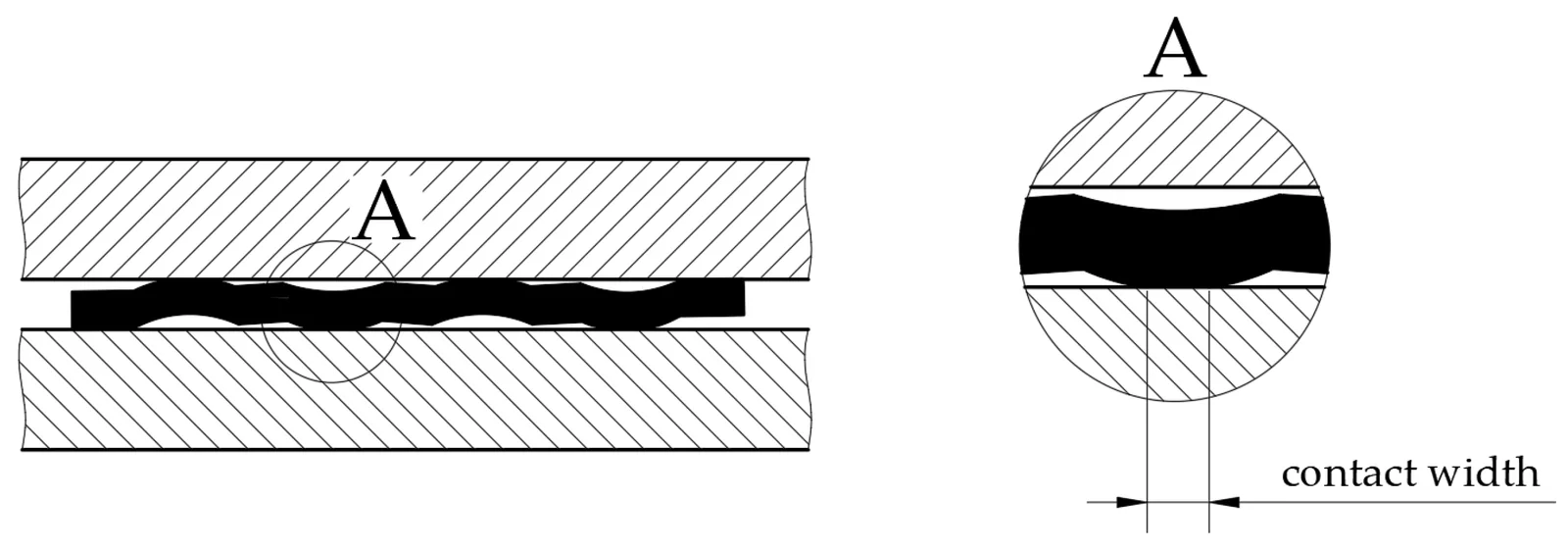
Contact geometry in a flanged joint with a corrugated gasket, based on [60].

By appropriately shaping the gasket cross-section, it is possible to influence the intensity of contact pressure and its location on the mating surfaces. Designs with a broad nominal contact area lead to a more widely distributed contact pressure, whereas gaskets with rounded, profiled, or locally protruding sealing regions enable contact pressure concentration in narrow contact zones. One of the most commonly used solutions for concentrating contact pressure involves gaskets that employ line contact or narrow annular contact zones. Saeed et al. [56] investigated a new corrugated metal gasket in which circumferential protrusions form sealing lines with the flange surfaces (Figure 4). Leak-tightness tests confirmed that appropriate shaping of the gasket geometry affects the contact pressure and contact area and, consequently, its sealing performance. Nurhadiyanto et al. [57] continued research on this type of gasket by analyzing the relationship between contact width, contact pressure, and leakage rate. Corrugated gaskets have the advantage of forming several concentric contact zones. The individual sealing lines may act as successive barriers to fluid flow, so a local discontinuity in one zone does not necessarily create a continuous leakage path across the entire sealing width. At the same time, the corrugated geometry provides relatively high compliance and elastic recovery, enabling high local contact pressures to be achieved with a lower tightening force and reducing the effects of bolt-load loss, including bolt loosening [58,59]. Their effectiveness, however, depends on the appropriate selection of geometric parameters, particularly the height and number of sealing lips, their pitch and overhang, and the gasket thickness, because these parameters affect the elastic response, contact pressure, and contact area [56].

A further development of the concept of contact pressure concentration in narrow contact zones is represented by designs using sharp-edged sealing rings. Jaszak et al. [61] analyzed fluid permeability at a metal-to-metal interface for a sharp-edged gasket described in patent [62], whose sealing surface consists of concentrically arranged sharp ridges forming contact lines with the flange surfaces. The resulting sealing mechanism is based on the local indentation of the sharp ridges into the rough surface of the mating components, which leads to gradual closure of the gaps and disruption of the continuity of leakage channels. Sharp-edged geometries concentrate contact pressure and may therefore permanently modify the mating surfaces. Because this modification can alter the contact conditions after the first loading cycle, leakage performance during unloading, reassembly, and cyclic loading cannot be inferred from the initial loading response and has not yet been systematically evaluated.

A similar mechanism of increasing local contact pressures occurs in metal lenticular gaskets analyzed by Zhou et al. [63]. Their analysis of contact and leakage in flanged joints with this type of gasket showed that joint tightness is affected not only by local contact pressures and the width of the real contact zone, but also by the contact diameter, which defines the position of the annular contact zone. An increase in contact diameter led to increased leakage. The effects of the analyzed parameters on leakage were evaluated analytically, and further experimental studies could help confirm the observed relationships.

**Figure 5:**
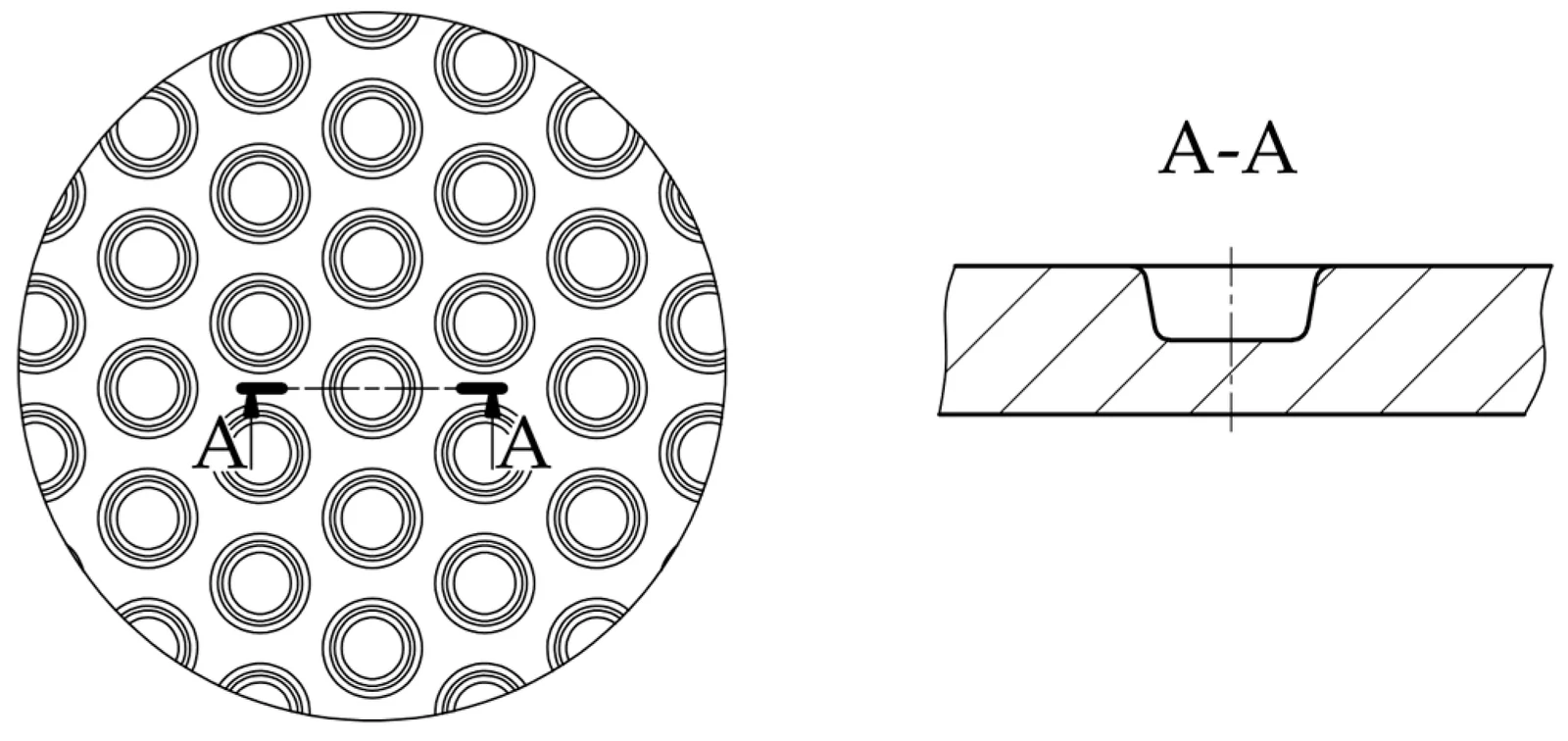
Variant of the sealing surface texture according to the patent by Ledrappier et al., based on [28].

Another direction in the development of sealing surface geometry involves designs that use a controlled microstructure of the contact surface. Ledrappier et al. [28] introduced a metal gasket with a textured sealing surface containing an array of microcavities (Figure 5). A characteristic feature of the proposed structure was the use of closed, mutually unconnected cavities that do not form continuous flow paths. The concept is intended to enable a simultaneous increase in local contact pressures while maintaining a relatively wide sealing zone capable of compensating for geometric deviations of the surface. The patent also presents comparative leakage results suggesting improved sealing performance of the textured surfaces relative to an untextured reference; however, the test methodology, repeatability, and measurement uncertainty are not described. This approach indicates a transition from the classical design of gasket macrogeometry toward the deliberate shaping of contact surface microgeometry to control the mechanism of leakage channel formation.

However, evidence on deliberately engineered sealing surfaces remains limited, and the relative capabilities of different manufacturing routes have not been systematically assessed. Future studies should therefore examine manufacturing and surface-modification methods, including controlled texturing, precision machining, and additive manufacturing, as tools for tailoring sealing surfaces to improve sealing performance.

Studies show that sealing performance in sealed joints with metal gaskets depends not only on gasket geometry, but also on the topography of the mating surfaces. Even in the case of geometries that provide high local contact pressures, real contact occurs only within microscale surface irregularities, while the continuity of leakage channels is largely determined by the topography of the sealing and mating surfaces. Accordingly, gasket geometry and surface topography should be evaluated jointly, because the contact conditions associated with gasket geometry interact with microscale surface irregularities to influence sealing performance. The following subsection presents studies on the influence of surface topography on sealing performance in sealed joints with metal gaskets.

### 3.2. Influence of Surface Topography on Sealing Performance

In sealed joints with metal gaskets, leakage is directly related to the topography of the mating surfaces. The character of machining marks, the directionality of irregularities, roughness, and the geometry of valleys and peaks determine both the real contact area and the possibility of continuous fluid flow channels forming. In contrast to gaskets manufactured from compliant materials, in which surface irregularities may be partially compensated for by deformation of the sealing material, even small irregularities in metal gaskets may lead to the persistence of microgaps responsible for leakage. For this reason, not only the height of the roughness profile is important, but also the directionality of the surface geometric structure resulting from the applied manufacturing process.

In engineering practice, one of the most commonly used measures of the averaged height of the roughness profile is the Ra parameter, which describes the arithmetic mean deviation of the profile from the mean line [64]. An increase in Ra is usually associated with an increase in the height of surface irregularities, which may promote the formation of larger fluid flow gaps and, consequently, lead to increased leakage. Such an effect was observed, among others, by Yang et al. [65] and Karohika and Haruyama [66]. It should be noted, however, that the Ra parameter does not describe the spatial arrangement of surface irregularities; therefore, its interpretation in the context of sealing performance requires the consideration of other topographic features as well, such as the directionality of the structure resulting from the applied manufacturing technology. Although Ra may correlate with leakage for some surface finishes, it does not account for the orientation and continuity of machining marks and is therefore insufficient as a standalone descriptor.

One of the earlier studies on the effect of surface manufacturing on the leak tightness of joints was presented by Arghavani et al. [26]. The work compared the leak tightness of flanged joints with flange sealing faces obtained by grinding, turning, and milling. Although the study did not concern all-metal gaskets, but instead PTFE, graphite, and graphite-filled spiral-wound gaskets, it remains relevant because it directly examined the effect of flange surface characteristics resulting from manufacturing on interfacial leakage. The results revealed that the Ra parameter is not always a sufficient indicator for assessing joint leak tightness. For ground surfaces, a clear relationship was observed between increasing roughness and increasing leakage intensity, whereas for turned and milled surfaces, the character of machining marks and the presence of radial channels enabling fluid flow were of greater importance. These observations emphasize that surface form and the geometry of machining marks may play a greater role in the leakage mechanism than the average surface roughness value alone. Overall, the relationship between Ra and leakage depends on the surface manufacturing process and gasket stress level, while the overall influence of surface characteristics also varies with gasket type.

The effect of the directionality of machining marks on the leakage mechanism was also investigated by Nitta et al. [67,68], who analyzed the real contact area in joints with a flat metal ring gasket manufactured from copper and mating with steel flanges whose surfaces were machined by turning. To visualize the contact regions, a 1 μm thick polymer film was placed between the gasket and flange surfaces before the joint was clamped, causing the contact traces to be replicated on the film. The tests were conducted for selected nominal contact pressure values in the range of 5–100 MPa. The replicated contact patterns revealed that, despite nominally flat contact, real contact occurs only at the peaks of the surface irregularities. As the load increased, the proportion of the real contact area increased, reducing the continuity of leakage channels. Two principal directions of possible fluid flow through the sealing zone were identified: the radial direction, perpendicular to the machining marks, and the circumferential direction, consistent with the direction of the grooves formed during turning. With further loading, the flow channels in the radial direction were gradually closed, whereas leakage in the circumferential direction persisted even at higher contact pressures. This behavior confirms that contact geometry cannot be analyzed solely at the nominal scale and provides direct experimental evidence that the spatial distribution and continuity of noncontact regions are important for leakage. However, the polymer film method does not directly measure the local height of the remaining microgaps, which also influences the leakage rate.

The importance of surface anisotropy after turning was also emphasized by Robbe-Valloire and Prat [69], who analyzed the microgeometry of face-turned surfaces using profilometric measurements and a statistical model. Their analysis identified a regular structure of spiral grooves resulting from the motion of the cutting tool. Consistent with the contact patterns identified by Nitta et al., their model indicated that this topography may lead to the formation of two types of leakage paths: circumferential paths running along the spiral valleys and radial paths formed locally as a result of contact discontinuities at the peaks of irregularities. Sealing performance was found to depend not only on the roughness value, but also on the height distribution of peaks and valleys. Contact discontinuities in the radial direction are particularly unfavorable because they may form short flow paths through the sealing zone. In turn, variations in valley height may promote blockage of circumferential leakage channels if local segments of the valleys are closed under the applied contact pressure. However, the model neither predicted the total leakage rate for a specific joint nor was experimentally validated. The analysis also assumed Gaussian peak and valley distributions, sinusoidal interpolation, and fully plastic asperity deformation. Therefore, its quantitative applicability to actual sealing joints remains limited.

The effect of the orientation of machining marks and the regularity of surface topography was analyzed in detail by Gu et al. [29]. A significant dependence of sealing performance on the orientation of grinding marks relative to the direction of fluid flow was observed. Machining marks parallel to the leakage direction promoted the formation of continuous flow channels, resulting in an increased leakage rate, whereas perpendicular marks had a beneficial effect by reducing the leakage rate. Surfaces with irregular and discontinuous topography, as well as surfaces with concentric marks, promoted a reduction in fluid leakage, whereas surfaces with a regular, radially oriented groove pattern exhibited the opposite effect. At the same time, an increase in the leakage rate was observed with increasing surface roughness, described by the Ra parameter. However, the study concerned hydraulic-oil leakage through a glass–aluminum interface rather than a metal-gasketed joint and used contact pressures of 0.01–0.07 MPa, well below those typical of practical static sealing applications. Thus, the results support the qualitative importance of machining-mark orientation and continuity, but the measured leakage rates are not directly transferable to metal-gasketed joints.

Extending this analysis, Shereef and Dhanish [30] evaluated the effects of different components of surface topography on leakage in a static metal-to-metal seal. The investigation considered the contact between a stainless steel valve with a ground surface and a polished valve seat, and analyzed parameters describing roughness, waviness, and form error in both 2D profile-based and 3D surface-based approaches. Among the investigated features, form error, particularly the flatness deviation of the valve surface, was more strongly correlated with leakage than roughness and waviness because it promoted extensive noncontact regions and continuous fluid flow channels. Roughness was also significantly correlated with leakage, whereas waviness had no significant effect within the investigated range. Comparison of the two characterization approaches further showed that 3D surface parameters enable more effective leakage prediction than 2D profile parameters, because they better represent the spatial character of microgaps in the contact zone. Because only ground valve surfaces and one valve-seat configuration were investigated, the reported relative importance of form error, roughness, and waviness should not be assumed to apply to all manufacturing processes or gasketed joints.

Taken together, the reviewed studies support the conclusion that sealing performance cannot be characterized by a single topographic parameter. Ra quantifies the mean roughness amplitude but does not describe the spatial arrangement or connectivity of noncontact regions. Directional machining marks may promote or interrupt continuous leakage paths, whereas form deviations may generate extended noncontact zones that persist under load. The relative influence of these surface features depends on gasket geometry, material compliance, contact pressure, manufacturing-induced anisotropy, and the orientation of the surface texture relative to the leakage direction.

Table 1 presents a summary of selected experimental studies analyzing the effect of surface characteristics on leakage in joints with metal gaskets. The studies included in the table were selected from the identified literature to represent experiments in which both contacting surfaces were metallic. The purpose of this comparison is to illustrate how gasket material, surface topography characterized by roughness parameters, and the surface manufacturing method influence leakage as a function of the applied load or contact pressure. The reported leakage values are not directly comparable because of differences in joint geometry, materials, test fluids, loading and pressure conditions, and the units used. The table indicates the types of gaskets, surfaces, roughness parameters, and contact conditions that have been considered to date in studies on the sealing performance of this type of joint.

Based on the review of studies on the effect of surface characteristics on joint sealing performance, it can be concluded that leakage in sealed joints with metal gaskets results from the simultaneous interaction of contact geometry, material properties, the magnitude of the applied load, and the topography of the mating surfaces. These factors affect the real contact area, the distribution of contact pressure, and the geometry of microgaps that may form connected fluid flow channels. Thus, neither surface characterization alone nor analysis limited to the nominal gasket geometry is sufficient for a complete description of sealing performance.

The reviewed experimental studies reveal two important limitations and research needs. First, substantial differences in joint geometry, materials, test conditions, methods of surface characterization, and leakage units prevent direct comparison of the reported results and limit the formulation of generally applicable relationships. Several relevant studies also concern joints with relatively compliant gaskets, non-gasketed metal-to-metal seals, or model interfaces formed by dissimilar materials rather than joints with solid metal gaskets. These studies help identify the mechanisms governing leakage, but their quantitative results cannot be directly generalized to all joints with metal gaskets. The comparability of available data is further limited by the relatively infrequent use of standardized leakage-testing procedures, as many studies employ custom test rigs and independently defined experimental procedures. Greater use of standardized testing methods would improve the repeatability and comparability of leakage measurements and facilitate their application in engineering practice. Future studies should therefore place greater emphasis on procedures consistent with relevant international and industrial standards, such as EN 13555 [53], ASTM F2836 [70], ASME B16.20 [54].

Second, experimental studies provide information on the effects of macroscopic loading conditions, contact conditions, and selected surface characteristics on leakage rates, but they generally do not provide a detailed quantitative characterization of the dimensions and continuity of microgaps formed at the sealing interface. In addition, many studies report only Ra or a limited set of profile parameters without characterizing the three-dimensional connectivity of valleys and noncontact regions. Consequently, experimental evidence alone is insufficient for reliable leakage prediction across different joint configurations. Addressing these limitations requires modeling approaches that couple the contact mechanics of rough surfaces with fluid flow through the resulting microgaps, as discussed in the following section.

## 4. Contact and Leakage Modeling

**Figure 6:**
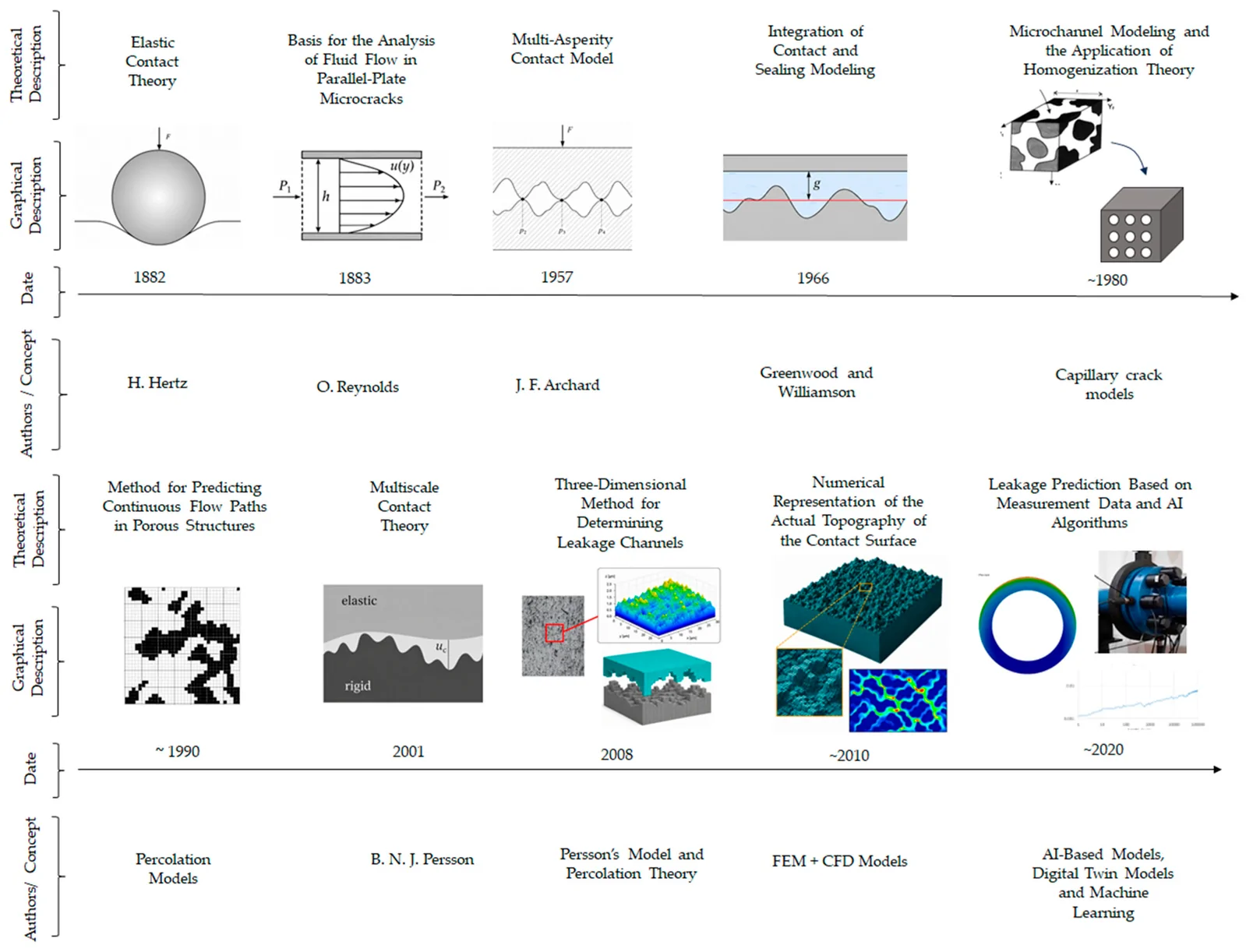
Chronological development of contact and leakage prediction models.

Modeling leakage from sealed joints requires a simultaneous description of the contact mechanics of rough surfaces and fluid transport in the microgaps formed at their interface. Over the years, numerous models have been developed to describe contact and leakage between rough surfaces, differing in their level of complexity, assumptions, and range of applicability. The chronological development of selected contact and leakage modeling approaches discussed in this review is summarized in Figure 6. The foundations of modern leakage modeling at rough interfaces can be traced to two complementary developments in the late 19th century: Hertzian elastic contact theory [71] and Reynolds’ description of viscous flow through a narrow gap between surfaces [72]. These concepts established the basic descriptions of surface contact and fluid flow that were subsequently extended to rough sealing interfaces.

Further developments focused on increasingly realistic representations of rough-surface contact and the geometry of potential leakage paths. Archard [73] introduced a multi-asperity description of rough-surface contact. Subsequently, contact models were developed that were later applied to the description of rough-surface contact for leakage analyses. In parallel, the capabilities of leakage modeling expanded through the use of simplified microchannel and capillary representations, homogenization concepts, and, later, percolation-based descriptions. The subsequent development of multiscale contact theory and three-dimensional methods further improved the representation of contact conditions and leakage paths across different length scales.

The beginning of the 21st century brought rapid development of numerical methods, both in contact modeling using the finite element method (FEM) and in the analysis of flow through microgaps using computational fluid dynamics (CFD). These approaches enabled increasingly detailed representation of actual surface topography, contact conditions, and leakage paths. More recently, there has also been a marked increase in the use of artificial intelligence and machine learning techniques, together with the development of digital twin concepts. Combined with experimental measurement data, these approaches can support the development of predictive models for leakage estimation.

The following subsections discuss the principal and most frequently cited contact and leakage modeling approaches in greater detail, with particular emphasis on their application to metal-to-metal sealing interfaces.

### 4.1. Modeling of Contact Between Rough Surfaces

**Figure 7:**
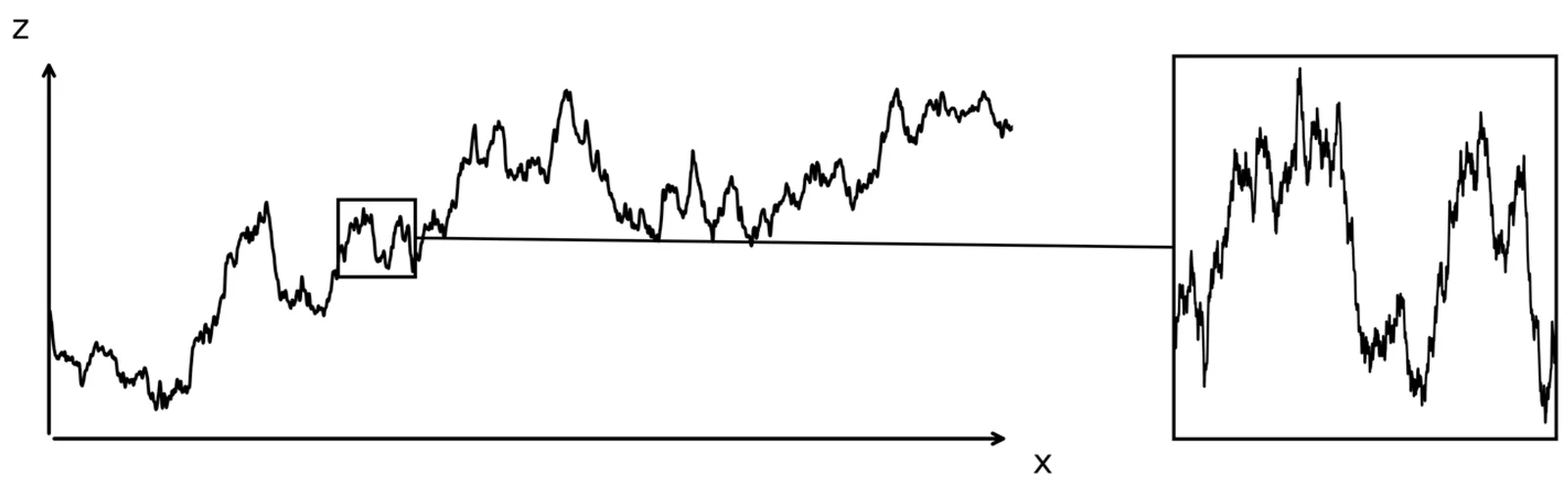
Multiscale nature of roughness, based on [74].

A theoretical description of contact between surfaces requires a precise characterization of the surface geometric structure, or microgeometry, of rough surfaces. In practice, nearly all macroscopic bodies, even those that appear smooth, exhibit roughness, and this roughness is multiscale in nature, i.e., it occurs over many length scales, from micrometers to nanometers (Figure 7). Consequently, real contact occurs only in small, randomly distributed regions, and its area typically represents only a very small fraction of the nominal contact area. Contact regions can be interpreted as small areas in which the irregularities of one surface are pressed against the irregularities of the other surface, undergoing elastic and/or plastic deformation. The multiscale and heterogeneous nature of contact makes the actual distribution of contact regions and the geometry of potential flow paths, i.e., leakage channels, in a gasketed joint complex and difficult to describe unambiguously.

Contact models for rough surfaces have already been discussed in several studies. Persson [75,76] primarily discusses his own multiscale formulation of contact mechanics. The review by Ghaednia et al. [77] provides a systematic overview of elastic-plastic microcontact models. Taylor et al. [78] discuss rough-surface contact models from the perspective of tribological applications.

To organize the discussion, the rough-surface contact models considered in this review are grouped according to their theoretical basis and modeling approach. Section 4.1.1, Section 4.1.2 and Section 4.1.3 present asperity-based, fractal, and spectral contact models, respectively. Section 4.1.4 discusses extensions of existing rough-surface contact models that account for more realistic contact conditions, while Section 4.1.5 focuses on advanced computational approaches. The reviewed models are then critically compared in Section 4.1.6 in terms of their assumptions, capabilities, computational requirements, and applicability to sealing problems. Finally, Section 4.1.7 summarizes the main limitations of current rough-surface contact modeling approaches and identifies remaining research gaps.

#### 4.1.1. Asperity-Based Contact Models

One of the earliest descriptions of contact between two bodies with parabolic surfaces pressed against each other by a normal force is the theory of frictionless elastic contact proposed by Hertz in 1882 [71]. Hertzian contact theory became a foundation for subsequent rough-surface contact models. In 1957, Archard [73] extended the description of elastic contact to the case of a rough surface in contact with a flat surface, accounting for the multiscale nature of irregularities and representing the surface as an array of spherical roughness asperities, forming multipoint contact.

In turn, Greenwood and Williamson [79] proposed in 1966 a statistical approach to the contact between a rough surface and a smooth surface, in which roughness is approximated by a set of spherical asperities with constant curvature and a Gaussian height distribution. This framework was extended by Greenwood and Tripp [80] to the case of contact between two rough surfaces. Subsequently, Bush, Gibson, and Thomas [81] used ellipsoidal paraboloids to describe rough surfaces, resulting in elliptical contact regions instead of the circular regions characteristic of the Greenwood-Williamson model. This modification provided a more accurate representation of the real contact area.

The models discussed so far were based primarily on an elastic description of microcontact, whereas the possibility of plastic deformation was accounted for mainly through criteria and indices defining the range of contact pressure acting on the microstructure. To account explicitly for local yielding, Chang et al. [82] introduced an elastic-plastic contact model for rough surfaces, in which the Hertz solution represents the limiting case for the elastic regime, while at higher contact pressures local plastic deformation of asperities is taken into account. Kogut and Etsion [83] presented a further development of this approach, providing a more accurate description of the transition regime between elastic and plastic contact.

Despite their physical transparency and relatively low computational cost, asperity-based models rely on idealized representations of surface microgeometry, including prescribed asperity shapes and statistical height distributions. In addition, interactions between neighboring asperities and the spatial connectivity of contact and noncontact regions are generally represented only in a simplified manner. These limitations are particularly relevant to sealing problems, in which fluid leakage depends not only on the real contact area but also on the formation of continuous noncontact paths across the sealing interface.

#### 4.1.2. Fractal Contact Models

An important advance in surface contact modeling was the use of the Weierstrass-Mandelbrot fractal function to describe roughness, as proposed by Majumdar and Bhushan in 1991 [74]. This approach made it possible to describe surface roughness using scale-independent fractal parameters, in contrast to earlier models based on statistical parameters, such as root mean square height, slope, or curvature, which depend on the adopted observation scale.

Nevertheless, the Majumdar-Bhushan model contains assumptions that are inconsistent with experimental observations; namely, it predicts that all microcontacts with an area smaller than the critical area are in the plastic regime, whereas, as the load increases, plastically deformed regions coalesce, the contact area increases, and a transition from the plastic to the elastic regime occurs.

**Figure 8:**
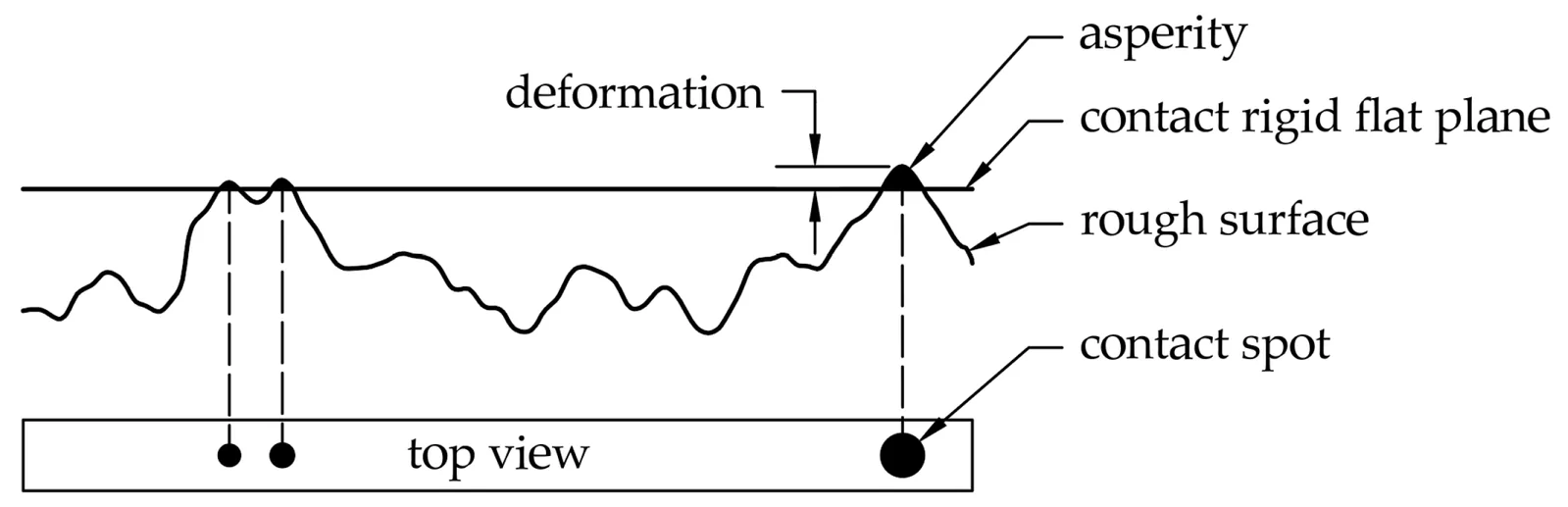
Contact between surfaces in the Majumdar-Bhushan model, based on [84].

In response to this inconsistency, Morag and Etsion [84] proposed a modification of the Majumdar-Bhushan model (Figure 8) at the level of a single asperity, eliminating the problem of an incorrect transition from the plastic to the elastic regime with increasing load. Miao and Huang [85] subsequently extended this approach to the level of the entire fractal surface. A later modification by Yuan et al. [86] addressed the transition from elastic to plastic deformation with increasing load in greater detail.

Using a modified Weierstrass-Mandelbrot function, Yu and Chen [87] generated three-dimensional rough surfaces and developed a semi-analytical elastic-plastic contact model for three-dimensional self-affine fractal surfaces. The fractal framework was later extended by Chen et al. [88] to account for multiscale effects in greater detail.

Relative to asperity-based models, fractal formulations offer a more natural description of roughness across multiple length scales, which is advantageous when small-scale surface features contribute to the formation of contact and noncontact regions. Nevertheless, their predictions depend on the identified fractal parameters and on the adopted wavelength range, so the apparent scale independence of the formulation does not eliminate sensitivity to surface characterization.

#### 4.1.3. Spectral Contact Models

Persson [24,89,90] proposed a different approach to modeling the contact of nominally flat surfaces, based on the random nature of the roughness profile, the description of roughness in the spectral domain, and the analysis of contact at successive length scales. This formulation enables determination of the contact pressure distribution and the real contact area as functions of load without introducing a single characteristic curvature of asperities. Moreover, it provides a consistent description of the evolution of the real contact area with increasing load and allows the transition from the elastic to the elastic-plastic regime to be taken into account.

This spectral framework was later extended by Tiwari and Persson [91] to line contact between a cylinder and a randomly rough plane, analyzing the effects of roughness and load on the nominal contact pressure distribution.

In contrast to asperity-based models, which represent roughness as a population of individual asperities with prescribed geometrical and statistical properties, and fractal models, which characterize its multiscale nature through fractal parameters, Persson’s approach describes the surface in terms of roughness components acting at different length scales and follows the evolution of contact as progressively finer scales are resolved. This formulation avoids the need to define individual asperity shapes or a characteristic asperity curvature and offers an alternative framework for describing surfaces exhibiting roughness over a broad range of length scales.

#### 4.1.4. Extensions of Rough-Surface Contact Models

The models discussed above remain the foundation of contact mechanics; however, modern approaches complement, develop, and extend their range of applicability. One recent trend in rough-surface contact modeling is the use of multiscale models describing contact under loading and unloading conditions, as presented, among others, in the studies by Yuan et al. [92] and Zhang et al. [93].

Rough-surface contact models that account for the curvature and macrogeometry of the mating components, rather than only nominally flat surfaces, are also being developed increasingly often, as exemplified by the studies of Chen et al. [94,95].

Such extensions improve the representation of specific features of real contacts, including loading history and surface macrogeometry, but they generally require a richer set of input data describing the relevant surface, geometric, and material characteristics.

#### 4.1.5. Advanced Computational Approaches

An alternative to analytical and semi-analytical contact models for rough surfaces is provided by discretization-based numerical models, which have been developed with increasing computational power, including the finite element method (FEM). Numerous examples of such approaches can be found in the literature, including the studies by Kartini et al. [96], Zhang et al. [97], and Dai et al. [98]. However, detailed numerical representation of rough-surface contact remains associated with high computational costs, especially when a large number of surface irregularities must be resolved.

The computational requirements become particularly important for multiscale rough surfaces because resolving the smallest relevant roughness scales requires a correspondingly fine spatial discretization. Consequently, direct numerical representation of roughness over a broad range of scales may become impractical when contact has to be analyzed over the entire sealing interface or repeatedly evaluated under different loading and surface conditions.

One approach to reducing this computational burden was presented by Yastrebov et al. [99], who proposed a reduced contact model based on information obtained from detailed finite element analyses of individual asperity contacts. This approach enables selected contact quantities to be determined without performing a full discretization-based simulation of the entire rough interface, thereby substantially reducing computational effort. Other strategies aimed at improving computational efficiency include multiscale approaches, homogenization-based contact models, and physics-informed data-driven methods. Examples include the approaches proposed by Carvalho et al. [100], Yang et al. [101], and Zhou and Song [102].

Further development of reduced-order and hybrid models represents a promising direction for rough-surface contact modeling. Reduced-order approaches may provide computationally efficient approximations of high-fidelity contact solutions, whereas hybrid models could combine detailed numerical analysis with analytical, homogenized, or data-driven descriptions at selected scales or regions. Such approaches have the potential to balance computational efficiency and predictive accuracy, which is particularly important for multiscale analyses of large sealing interfaces and for coupling contact calculations with leakage models. Their further application will require careful assessment of how model reduction affects the prediction of local contact quantities relevant to the formation and connectivity of leakage paths.

#### 4.1.6. Comparative Assessment of Rough-Surface Contact Models

In general terms, microscale contact modeling involves calculating the unit contact area formed by a characteristic asperity of the roughness profile and the counter-surface with which it comes into contact, as well as calculating the force causing deformation of this asperity. A summary of selected rough-surface contact models discussed in this section is presented in Table 2.

A common feature of the models summarized in Table 2 is their aim to determine the relationship between load and real contact area; however, they differ in how surface irregularities are represented and in the range of deformation mechanisms considered. The Hertz model provides a fundamental solution for a single elastic contact and is therefore most suitable as a local description of the contact of an individual asperity. This formulation does not account for the multiple and random nature of contact between rough surfaces. In addition, Hertzian theory is limited to the elastic regime and therefore cannot directly describe local plastic deformation that may occur under the high contact pressures characteristic of metal-to-metal contact. In the modeling of joints with metal gaskets, Hertzian theory can be used in multiscale models to describe contact at the macroscopic scale, but its application under conditions involving yielding requires an extension that accounts for elastoplastic or plastic deformation.

The Archard model extends the Hertzian concept to multiple contacts and shows that the relationship between real contact area and load depends on whether increasing load causes new contact regions to form or existing contact regions to expand. Because of its idealized representation of asperity geometry, the model is primarily conceptual, and its applicability to the quantitative representation of specific real surface topographies is limited.

The Greenwood-Williamson model is more suitable for describing an entire rough surface because it enables the statistical determination of real contact area, load, and surface separation. Its primary limitation is the assumption that all contacting asperities deform elastically. Chang et al. [82] indicated that some asperities may exceed the elastic limit even at relatively low loads and that, at higher loads, the assumption of independent microcontacts may also cease to be valid. In this respect, the Chang-Etsion-Bogy model is more appropriate for metal-to-metal contact because it accounts for the coexistence of elastic and plastic deformation. Importantly, from the standpoint of sealing, comparison of the two models showed that differences in the predicted real contact area may be relatively small, whereas differences in surface separation are considerably greater [82]. This means that a model that accurately predicts real contact area does not necessarily predict the microgap heights relevant to leakage with comparable accuracy.

The Majumdar-Bhushan, Miao-Huang, and Yuan et al. models differ from asperity-based models primarily through their use of a fractal representation of the surface, which offers the advantage of accounting for the multiscale nature of roughness. However, the original Majumdar-Bhushan model exhibits an important physical inconsistency because it predicts a transition from plastic to elastic contact as the contact size increases. This issue was corrected in later models through the use of scale-dependent criteria for transitions between deformation regimes. The Miao-Huang model retains the fractal representation of the surface while restoring a transition sequence consistent with classical contact mechanics, whereas the model of Yuan et al. additionally distinguishes elastic, elastoplastic, and fully plastic regimes. These models are therefore more suitable for analyzing surfaces for which both the multiscale nature of roughness and changes in deformation behavior with increasing load are important.

Greater model complexity, however, does not automatically result in greater accuracy over the entire load range. Comparisons reported in [74,86] indicate that fractal models may provide better agreement within one load range, whereas asperity-based models may show smaller deviations under other conditions. Model selection should therefore depend on the dominant deformation regime, the characteristics of the surface, and the contact quantity required for subsequent analysis rather than solely on the level of model complexity.

Analytical and semi-analytical models are particularly useful for describing surfaces with a random character because they allow averaged contact quantities to be determined from statistical or fractal parameters without explicitly resolving every asperity. Numerical models, in contrast, enable a more detailed analysis of a specific surface topography, including local distributions of contact pressure, deformation, and contact regions. Their main limitation is the high computational cost, which increases with the resolution of the surface representation and the size of the analyzed area.

#### 4.1.7. Limitations and Research Gaps in Rough-Surface Contact Modeling

Despite substantial progress in rough-surface contact modeling, several important areas still require further investigation. These include, in particular, the limited validation of models for real metal surfaces under contact pressures representative of sealing joints and the simplified representation of surface topography in some models, especially with respect to surface anisotropy and directionality. Another important issue is the treatment of loading history and the permanent evolution of surface topography during unloading, reloading, and successive contact cycles. From the standpoint of leakage modeling, particular attention should also be given to linking predicted contact quantities with the spatial distribution and connectivity of microgaps that may form potential flow paths. At the same time, approaches are needed that can account for multiscale topography, macrogeometry, and material nonlinearities at a reasonable computational cost, while predicting both local and global contact characteristics without requiring full numerical resolution of all roughness scales.

In the analysis of gasketed joints, it is essential to describe not only the contact between the mating surfaces, but also the flow through microgaps remaining between rough surfaces. Contact modeling provides information on the geometry of the noncontact regions and on quantities such as the real contact area, local deformation, contact pressure, and gap dimensions, all of which influence the formation of potential fluid flow paths. Because fluid flow is highly sensitive to the dimensions of narrow gaps, even small differences in the predicted interface geometry may lead to substantial changes in the calculated leakage rate. Reliable contact modeling therefore provides the foundation for accurate sealing analysis and realistic leakage prediction. The following subsection is devoted to a review of leakage models.

### 4.2. Modeling of Leakage Through Contact Surfaces

Modeling leakage at the interface between rough surfaces can be formulated in three related stages:

1. modeling the contact;
2. describing the geometry of the flow gap;
3. calculating the flow through the gap.

Contact is modeled using the methods described in Section 4.1. The geometry of the flow gap, or leakage channels, is described, among others, using percolation theory, the critical constriction model, effective medium theory, and porous medium models. Depending on the adopted representation of the geometry, the flow, or leakage rate, is most often calculated based on the Poiseuille law, the Reynolds equation, Darcy’s law, the Navier–Stokes equations, or computational fluid dynamics (CFD). The following paragraphs present a review of studies on leakage modeling at the interface between rough metal surfaces.

To structure the review, the leakage models are grouped according to the surface configuration and the scale at which contact and fluid flow are represented. Section 4.2.1 considers leakage models for nominally flat rough surfaces, whereas Section 4.2.2 focuses on multiscale approaches that couple global contact conditions with local flow through microgaps. Section 4.2.3 addresses models developed specifically for turned surfaces, where the directional structure produced by machining plays an important role in the formation of leakage paths. Section 4.2.4 provides a comparative assessment of the reviewed leakage models, while Section 4.2.5 summarizes their principal limitations and identifies remaining research gaps.

#### 4.2.1. Leakage Models for Nominally Flat Rough Surfaces

One case analyzed in the literature is leakage at the interface between two rough, nominally flat metal surfaces. For this configuration, Persson [103] combined his own contact model, which accounts for plastic deformation, with Bruggeman effective medium theory to determine the leakage rate. By contrast, Feng et al. [104,105] used a modified Majumdar-Bhushan model to describe contact and determined the leakage rate based on the theory of laminar flow of an incompressible viscous fluid and fractal geometry theory.

A different fractal formulation was adopted by Zhang et al. [12,13], who represented the surface roughness profile using a three-dimensional Ausloos-Berman function [106], whereas contact was modeled using Yuan’s fractal model [86]. The leakage model was based on Yu’s fractal theory of fluid transport in porous media [107,108,109], in which the flow gap is represented as a bundle of tortuous capillary tubes with a fractal diameter distribution. A related approach by Liu et al. [110] used the Weierstrass-Mandelbrot function to represent surface roughness and their own fractal contact model [111] to determine the contact conditions. Leakage was then calculated using the fractal theory of flow through porous media.

Based on optical profilometer measurements of two rough surfaces, Cui et al. [112] developed a three-dimensional percolation grid model in the sealed interface region. Leakage was determined using a model based on porous media flow theory that accounts for effective porosity. A different numerical formulation was adopted by Lin et al. [113], in which surface roughness was described using a three-dimensional Weierstrass-Mandelbrot function, while FEM was used to model contact. The leakage description relied on percolation theory in porous media, with the porosity and permeability of the sealing interface being determined.

A different approach to leakage modeling was proposed by Gu et al. [114], who developed a model for predicting oil leakage in static seals that accounts for surface wettability by decomposing the pressure drop across the sealing interface into hydrostatic, microcapillary, and viscous contributions. This model extends the range of physical factors considered in leakage prediction.

Models for nominally flat rough surfaces differ primarily in how the flow gap and the physical mechanisms governing fluid transport are represented. Approaches based on effective medium theory, fractal models, or porous media flow allow the leakage rate to be determined without explicitly resolving the complete geometry of individual microgaps, thereby reducing computational cost. However, this simplification requires the actual structure of the flow channels to be represented by equivalent parameters, whose determination may require surface topography characterization, contact model parameters, and, in some cases, experimentally determined coefficients. From the standpoint of sealing performance, the ability to account for the connectivity of flow paths is particularly important. Therefore, percolation-based approaches are particularly useful when the mean gap height or porosity alone is insufficient to describe the connectivity of noncontact regions.

#### 4.2.2. Multiscale Leakage Models

In some studies, leakage modeling has been extended to account for both macroscopic contact conditions, including the distribution of contact pressure on the gasket surface, contact width, and the geometry and deformation of the gasketed joint, as well as microscale surface roughness.

**Figure 9:**
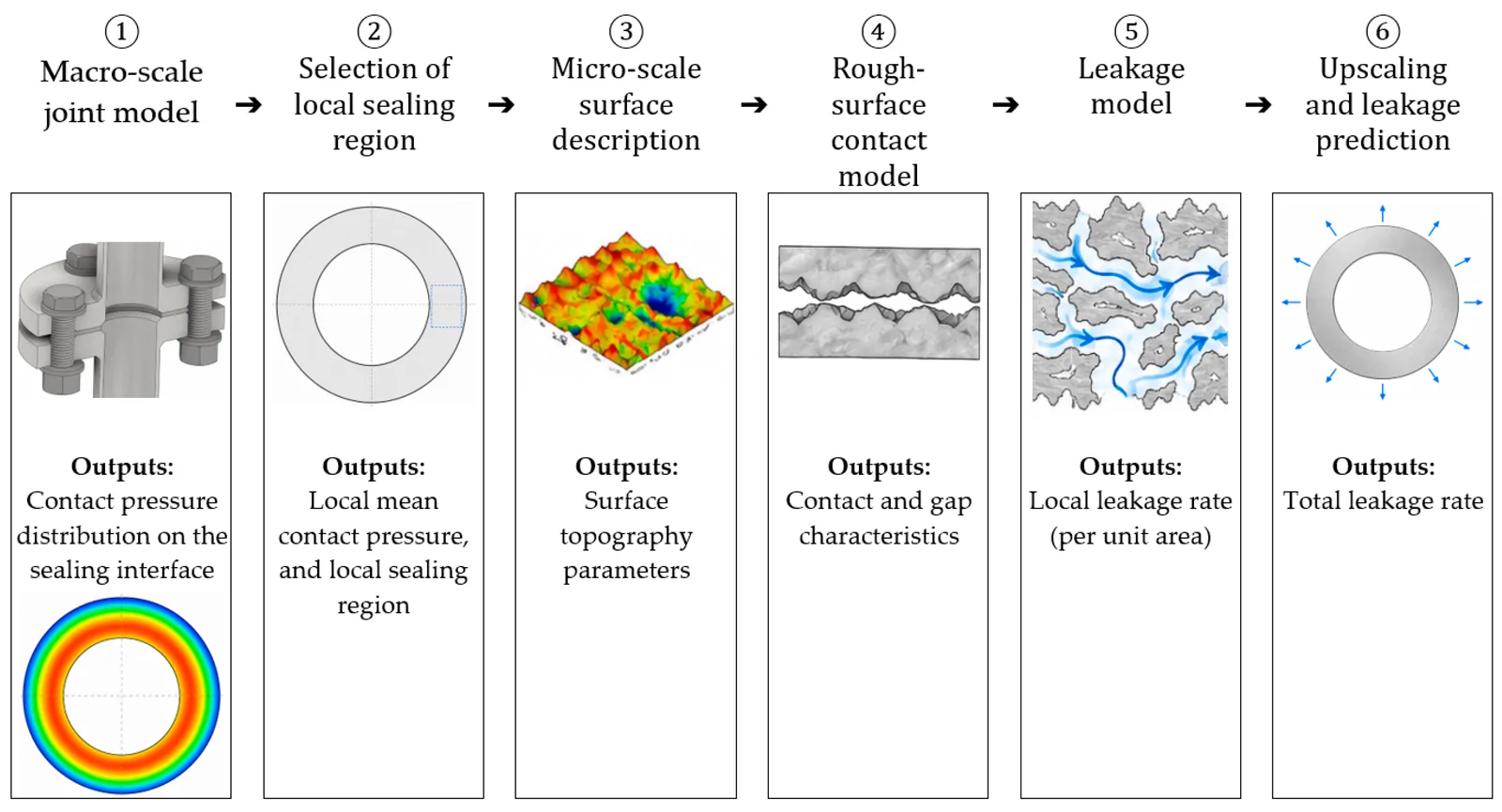
General multiscale workflow for leakage prediction at a metal-to-metal sealing interface.

A general multiscale workflow for leakage prediction at metal-to-metal sealing interfaces is illustrated in Figure 9. The analysis begins with a macroscale model of the entire joint to determine the contact pressure distribution and identify a local sealing region for further analysis. The surface topography within this region is then characterized at the microscale and used in a rough-surface contact model to determine local contact and gap characteristics. These results provide the input to the leakage model, from which the local leakage rate is determined and subsequently upscaled to predict the total leakage rate for the sealing interface.

In the multiscale model proposed by Zhang et al. [34], the macroscopic distribution of contact pressure, determined for the entire gasketed joint, served as a boundary condition for a finite element microcontact model based on the actual geometry of the sealing surface measured using a 3D profilometer. Based on the contact simulation results, the geometry of the flow gap was obtained, and the flow was calculated using CFD.

In a series of studies, Pérez-Ràfols et al. [31,32,33] progressively developed a stochastic two-scale leakage model. In this model, the global scale covers the entire sealing region and is used to determine the global fluid pressure distribution and total leakage, whereas the local scale represents surface roughness and enables determination of local permeability based on a detailed description of contact and flow in small regions, or domains. Coupling between the two scales is achieved through a stochastic element, which allows the random nature of the surface profile to be taken into account.

For a metal lenticular gasket, Zhou et al. [63] combined an analytical elastic-plastic contact model, enabling determination of contact stresses and contact width, with a microscale leakage model based on measurements of the actual surface. The microgeometry of the contact was reduced to a geometrically equivalent roughness profile described by the shape of regular wedges representing surface irregularities in three dimensions; these were then replaced with capillaries of the same volume, and the flow rate was determined for incompressible laminar flow based on the Navier–Stokes equations.

For a sharp-edged gasket, the model of Jaszak et al. [61] coupled FEM calculations representing the macroscopic deformation and indentation of the sharp gasket ridges on the sealed surfaces with an analytical description of flow through a porous medium. The roughness profile of the sealed surfaces was described using the Ausloos-Berman fractal function [106], whereas leakage was calculated using Darcy’s equation.

Another multiscale formulation, introduced by Zhang et al. [115], combines analysis of the entire gasketed joint with analysis of flow through microgaps at the surface interface. In this model, FEM analysis of the entire gasketed joint is used to determine the nonuniform distribution of contact pressure on the sealing surface. Then, using a microcontact model, local contact pressures are converted into local gap heights. The resulting local flow resistances are subsequently used to calculate total leakage using the flow resistance network method.

Multiscale approaches represent an important step toward more realistic leakage prediction because they allow the nonuniform contact pressure distribution resulting from the geometry and deformation of the entire gasketed joint to be taken into account and subsequently used to determine local contact and flow conditions. Compared with models based solely on an average contact pressure, these approaches preserve the relationship between the macroscopic state of the joint and the local geometry of the microgaps. However, the increased level of detail requires more input data and is associated with higher computational cost, particularly when local contact conditions are determined from measured 3D surface topography or FEM analyses. Another important limitation is the representation of surface microtopography. Simplified asperity geometries may not adequately represent surfaces with strongly directional structures, such as turned surfaces.

#### 4.2.3. Leakage Models for Turned Surfaces

**Figure 10:**
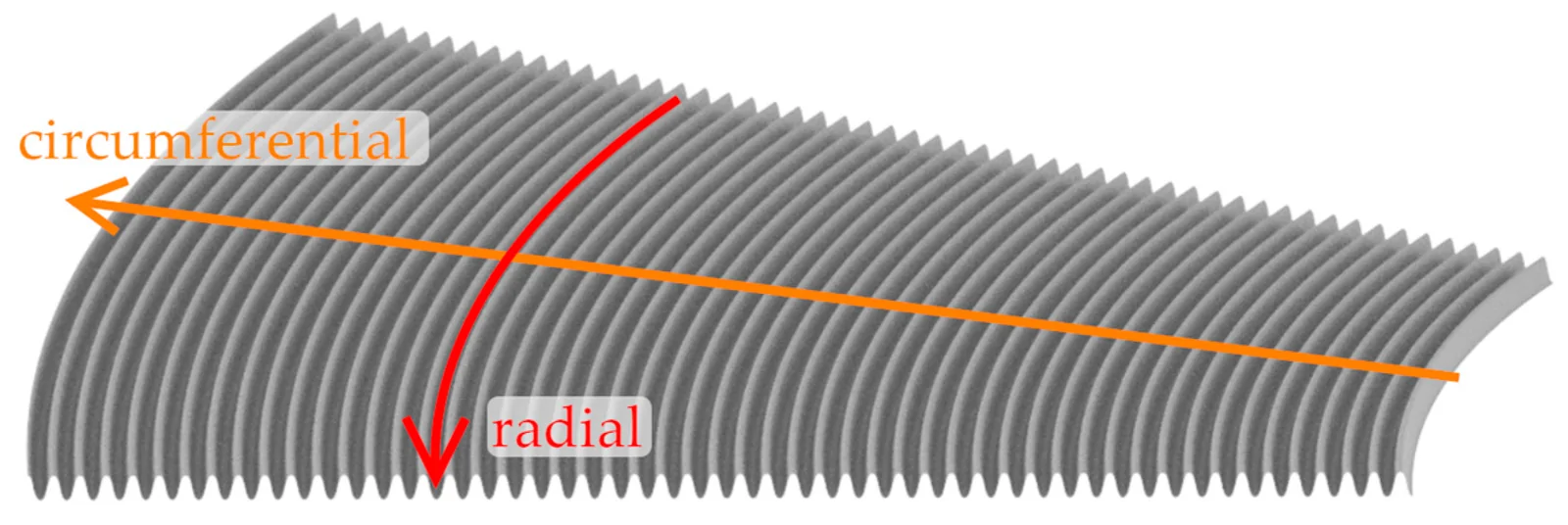
Visualization of a turned surface, based on [116].

A separate case is leakage modeling for turned surfaces, in which, in addition to random roughness, a directional surface structure associated with the presence of a spiral groove is also present. For a model metal ring gasket with a spiral groove representing a turned surface (Figure 10), Geoffroy and Prat [116] performed an analytical leakage analysis. Their analysis identified a possible sharp transition, with increasing load, from predominantly radial leakage, occurring through local passages between adjacent turns of the groove, to circumferential leakage along the spiral groove.

To account simultaneously for the spiral groove characteristic of turned surfaces and smaller-scale roughness, Pérez-Ràfols et al. [117] developed a numerical leakage model for metal-to-metal seals. The model coupled calculations of contact and surface deformation with calculations of flow through the gap based on the Reynolds equation. According to the resulting predictions, the leakage rate is determined not only by the proportion of real contact area, but also by the spatial distribution and interconnection of contact regions and flow gaps.

Models developed for turned surfaces account for a feature that is not represented by approaches based solely on a random description of surface roughness, namely the deterministic and strongly directional structure of the spiral groove. This makes it possible to distinguish between radial and circumferential flow mechanisms and to analyze changes in the dominant leakage path as the contact conditions vary. However, the specialized nature of these models limits their direct applicability to surfaces produced by other manufacturing methods, for which the directional structure and the mechanisms governing the formation of flow channels may differ.

#### 4.2.4. Comparative Assessment of Leakage Models

Selected leakage models used to describe flow through microgaps at the interface between metal surfaces are summarized in Table 3.

Across the leakage models summarized in the table, the predicted leakage rate is directly proportional to the pressure difference, consistent with the underlying physics of flow through the sealing interface. In several formulations, leakage also depends on the interfacial gap height, which can be determined, among other methods, from micromechanical contact modeling, and it varies inversely with fluid viscosity, which in turn depends on temperature and the type of sealed fluid.

In its basic form, Persson’s approach is based on the Bruggeman effective medium formulation and describes pressure-driven flow through microgaps formed within the surface microstructure while accounting for plastic deformation. Although initially formulated for a unidirectional surface structure, the approach can also be applied to interfaces with bidirectional, orthogonally oriented surface features. Fluid-pressure-induced surface deformation is neglected, and turbulent effects are not considered, although turbulence may occur under actual operating conditions, particularly at very high sealed-fluid pressures. The approach is therefore primarily applicable to viscous-fluid flow through very narrow gaps under predominantly laminar conditions, where the gap height is much smaller than its lateral dimensions and the fluid pressure is low relative to the nominal contact pressure. Another important aspect is the sensitivity of the predicted leakage rate to the scale at which the surface is characterized. As the resolution of the surface representation increases, the values of the characteristic dimensions L_x_ and L_y_ may change substantially, whereas insufficient spatial resolution may lead to a significant underestimation of leakage.

The formulation introduced by Feng assumes laminar flow of a viscous, incompressible fluid through the sealing interface. Its governing equation contains several coefficients that must be determined experimentally, which may limit its practical engineering applicability. These coefficients are related both to surface characterization, including the fractal dimension D, and to quantities derived directly from leakage measurements. In addition, the microchannels are represented by a regularly repeating sinusoidal geometry, which substantially simplifies the actual microgeometry of rough surfaces characterized by asperities of different heights and randomly distributed spatial densities. Validation was performed using an automated hydraulic press that applied contact pressures ranging from 62 to 250 MPa to a metal gasket. The annular specimen had an inner diameter of 40 mm and an outer diameter of 60 mm, while gas at pressures from 1 to 10 MPa was supplied to the chamber enclosed by the gasket and the loading plates. Leakage was determined using a differential method based on the pressure drop of the sealed fluid. However, no reference to a standardized test procedure was provided, and measurement uncertainty was not evaluated. Comparison of the experimental leakage measurements with the analytical predictions indicates that the maximum discrepancy exceeds 50% at lower fluid pressures and decreases as the pressure increases.

A different approach was adopted by Zhang, whose governing equation is based on flow through an annular porous medium and follows directly from Darcy’s law. The principal parameter is the permeability of the porous region formed by the space between the contacting surfaces. Its determination requires coefficients describing the fractal characteristics of the roughness profile. The associated Ausloos-Berman function enables different orientations of the surface structure to be represented, including orthogonal, irregularly oriented, and concentric patterns, the latter being characteristic of typical pipeline flange surfaces. Full definition of the model requires analysis of the roughness profile to determine the fractal dimension and scale coefficient. In addition, the parameter describing the mean pore diameter depends on the degree of penetration of the modeled rough layer into the smooth rigid counterface and is therefore strongly affected by the assumed deformation regime, whether elastic, elastoplastic, or plastic. The experimental results reported in the study show very good agreement with the analytical predictions. The approach appears particularly suitable for leakage prediction in relatively soft metal sealing materials, such as bronze and aluminum.

Unlike the previously discussed formulations, the approach proposed by Gu explicitly incorporates surface wettability. Leakage remains directly proportional to the pressure difference between the sealed-fluid side and the ambient side, but the overall pressure difference is divided into three components: hydrostatic pressure drop, pressure drop associated with microcapillary effects, and viscous pressure drop. Analytical descriptions of the latter two components make it possible to account for surface wettability, which was found to have a major effect on leakage reduction for oleophobic surfaces. The experiments showed that such surface modification could reduce leakage by as much as 64%. The resulting wettability-based theoretical formulation can also predict leakage for different roughness levels and contact angles. Validation was carried out using a custom experimental setup in which leakage was measured at the interface between a metal specimen and a glass plate. The glass plate enabled direct observation of oil propagation within the contact region. Rectangular aluminum specimens measuring 60 × 30 mm and 5 mm in thickness were prepared with three roughness levels of 6, 4, and 2 μm. Their contact surfaces were modified with perfluorodecyltrimethoxysilane to obtain different wettability conditions, while hydraulic oil with a dynamic viscosity of 8.5 mPa·s was used as the test fluid. Because of the limited strength of the glass plate, contact pressures were restricted to 0.01–0.07 MPa. Oil was supplied through a centrally located opening in the aluminum specimen. As expected, leakage increased with increasing surface roughness. More importantly, the oleophobic aluminum surfaces, with a contact angle of 142°, exhibited greater resistance to fluid flow through the microgap than the oleophilic surfaces, for which the contact angle was approximately 1°. As in several of the other studies, no uncertainty analysis was performed. Nevertheless, the experimentally measured and analytically predicted leakage rates showed good agreement over the investigated range of contact pressure, with the maximum deviation remaining below approximately 10%.

The Zhang approach also provides an example of a typical two-scale leakage modeling strategy. Its implementation first requires determination of the contact pressure distribution between the interacting surfaces at the macroscale. This distribution can be obtained numerically using FEM or experimentally using pressure-sensitive films or advanced pressure-mapping sensor arrays. The averaged contact stress is then transferred to the microscale, where the gap height is determined using a representation of surface asperities as hemispherical features uniformly distributed over a surface contacting a rigid metal counterface. Application of the approach therefore requires experimental or numerical contact data together with appropriate material properties. Experimental validation indicates that it can be effectively applied to metal surfaces. One limitation, however, arises from the assumed geometry of the modeled microstructure. The uniform distribution of asperities may not adequately represent strongly directional surfaces, particularly those produced by turning. Validation was performed using a custom test rig in which leakage was measured as a function of contact pressure between metal surfaces representing the sealing interface between a valve body seat and valve plug. The specimens were manufactured from stainless steel, and the sealing contact width was 10 mm. Test-fluid pressure ranged from 0.5 to 11 MPa. Although measurement uncertainty was not reported, comparison of the experimentally measured and analytically calculated leakage rates shows good agreement, with an approximate maximum deviation of less than 20%.

Overall, the models summarized in the table exhibit several limitations that may affect the predicted leakage rate. These include incomplete treatment of thermal effects, such as thermal expansion of the surface microstructure, as well as metal creep and time-dependent changes in material properties. No single model can therefore be regarded as universally applicable to all gasket types and operating conditions. Model selection should instead reflect the surface characteristics, material properties, joint geometry, and flow conditions relevant to the particular sealing system. Although the reviewed approaches generally show reasonable agreement with experimental measurements, they often require a substantial amount of input data and rely on simplified representations of surface microgeometry. Direct comparison of the individual models and their experimental and theoretical results is also difficult because the validation procedures differed considerably among the reviewed studies. Different materials and roughness levels were investigated, different contact pressures were applied, and different test fluids and fluid pressures were used. Despite these differences, the reported analytical and experimental results consistently show that leakage tends to increase with increasing surface roughness and fluid pressure and to decrease with increasing contact pressure.

#### 4.2.5. Limitations and Research Gaps in Leakage Modeling

Models that use a simplified representation of flow-gap geometry based on analytical or semi-analytical contact models, as well as simplified descriptions of flow through microgaps, can reduce computational cost, although often at the expense of predictive accuracy. Numerical methods such as FEM and CFD enable a more detailed representation of surface topography, including its anisotropy and directional characteristics, as well as of microgap geometry and fluid flow. However, resolving the smallest roughness scales is extremely challenging because of the very fine spatial discretization required and the associated computational demands, which may become prohibitively high or even practically unattainable. Therefore, the development of reduced-order, hybrid, and other computational approaches capable of maintaining relatively high predictive accuracy while substantially reducing computational cost appears to be a promising research direction.

Another important direction for future research is the reliable experimental validation of models, particularly multiscale models, in which simplifications or errors introduced at one scale may significantly affect the accuracy of leakage prediction at the system level. Validation should therefore include not only the final leakage rate but, where possible, also intermediate quantities such as the contact pressure distribution and local gap geometry. An additional limitation is the restricted comparability of available experimental results due to differences in materials, surface topography, loading ranges, test fluids, and experimental procedures. Moreover, many studies do not quantify the uncertainty associated with experimental measurements, whereas leakage models are generally formulated in a deterministic manner and do not account for uncertainty in their predictions. Therefore, greater attention should be given to the quantitative assessment of experimental uncertainty and to the development of probabilistic approaches to leakage prediction.

Another research gap is the limited use of advanced digital technologies, including machine learning (ML), artificial intelligence (AI), and digital twins (DT), for leakage prediction in joints with metal gaskets. Their application could enable faster processing of large datasets, identification of complex relationships among surface parameters, contact conditions, and leakage, as well as updating predictions based on experimental or operational data.

The review of leakage models at metal-to-metal interfaces shows that this area remains under intensive development. In more recent studies, there is a clear tendency to develop models for specific types of gaskets and gasketed joints, as well as to account for additional factors affecting sealing performance.

## 5. Future Perspectives

Based on the research gaps identified throughout this review, several directions for future research can be distinguished. Future research on the sealing performance of joints with metal gaskets should increasingly use detailed surface characterization to support the deliberate design of sealing surfaces. The reviewed studies indicate that individual profile parameters do not fully describe the spatial distribution, directionality, and continuity of surface irregularities that are relevant to the formation of microgaps and leakage paths. Therefore, greater use of actual three-dimensional surface topography measurements and advanced areal surface metrology should become an important research direction. At the same time, surface characteristics can be treated as an element of joint design rather than solely as a consequence of the manufacturing process. Controlled texturing, precision machining, manufacturing-process optimization, and additive manufacturing provide opportunities to shape sealing surfaces in ways that limit the formation of continuous flow channels.

A second major direction is the further integration of rough-surface contact modeling with leakage modeling. Reliable leakage prediction requires linking the macroscopic conditions resulting from the geometry, loading, and deformation of the entire joint with a microscale description of real contact and the geometry and connectivity of microgaps. Multiscale approaches are therefore particularly promising because they enable information to be transferred between the scale of the entire joint and the scale of local contact and flow phenomena. Their further development should also account for changes in contact conditions resulting from loading history and surface evolution. At the same time, the computational cost of such analyses must be reduced. Therefore, the development of reduced-order, hybrid, and other computationally efficient approaches capable of maintaining sufficient predictive accuracy without fully resolving all surface roughness scales remains an important direction.

Model development should proceed in parallel with reliable experimental validation. This is particularly important for multiscale approaches, for which validation should, where possible, include not only the final leakage rate but also intermediate quantities related to contact conditions and gap geometry. Greater comparability of experimental results is also needed through broader use of standardized testing procedures and relevant industrial requirements, including EN 13555, ASTM F2836, and ASME B16.20, as well as more consistent determination and reporting of repeatability and measurement uncertainty. A separate direction in modeling is probabilistic leakage prediction, which may complement the deterministic approaches currently used.

Further development of leakage prediction methods may also be supported by advanced digital technologies, whose application to joints with metal gaskets remains limited. Machine learning and artificial intelligence can support the analysis of large datasets and the identification of complex relationships among surface characteristics, contact conditions, and leakage, whereas digital twins may enable predictions to be updated based on experimental or operational data. Overall, the review indicates that a major direction for future development is the integration of advanced surface characterization and design with multiscale contact and leakage modeling, while simultaneously improving computational efficiency, validation reliability, and consideration of prediction uncertainty. Such an approach may provide a basis for more reliable sealing-performance prediction and for the design of sealing and mating surfaces aimed at improving joint tightness.

## 6. Conclusions

Overall, the reviewed literature indicates that sealing performance in sealed joints with metal gaskets results from the complex interaction of contact geometry, surface topography, material properties, and loading conditions. A complete description of this phenomenon requires simultaneous consideration of rough-surface contact mechanics and fluid flow through microgaps formed in the contact zone. Based on the synthesis of the reviewed literature, the following conclusions can be drawn:

- The combined evidence from experimental and modeling studies demonstrates that joint sealing performance is not directly determined by a single roughness parameter or by contact pressure alone. Of key importance is the geometry of the noncontact regions formed under load, particularly the dimensions and connectivity of microgaps capable of forming continuous flow paths. Gasket geometry primarily affects the distribution and concentration of contact pressure, whereas surface topography determines the local formation and closure of microgaps. Therefore, contact geometry and surface topography should be considered jointly, and surface assessment based solely on amplitude parameters, such as Ra, is insufficient for reliable evaluation of sealing performance.
- The review of experimental studies indicates limited comparability of the available results due to differences in test conditions and measurement procedures. An additional limitation is the incomplete reporting of repeatability and measurement uncertainty. This restricts the formulation of general relationships between surface characteristics and leakage, and also hinders reliable validation and assessment of the predictive capability of models.
- The critical comparison of contact and leakage models does not identify a single approach that is the most accurate and most appropriate for all surface types, materials, and operating conditions. The suitability of a model depends, among other factors, on how the surface geometric structure is represented, how deformation mechanisms are described, how the flow-gap geometry is represented, and how fluid flow is modeled. Moreover, increasing model detail does not automatically improve accuracy over the entire range of contact conditions. Model selection should therefore be based on the characteristics of the analyzed joint, the dominant deformation and flow mechanisms, and the purpose of the analysis rather than solely on model complexity.
- Among the approaches reviewed, multiscale methods appear to represent the most promising direction for modeling because they enable the behavior of the entire joint to be linked with local contact and flow phenomena. Further development of predictive methods should include the use of actual 3D surface topography, consideration of loading history and surface evolution, development of reduced-order models and hybrid approaches, probabilistic leakage prediction, and the use of machine learning, artificial intelligence, and digital twins.
- From the standpoint of improving sealing performance, an important direction is the deliberate design of sealing and mating surfaces. Advanced surface metrology, controlled texturing, precision machining, optimization of manufacturing processes, and additive manufacturing are particularly relevant in this context because they enable surfaces to be tailored to limit the formation of continuous leakage paths.

More reliable leakage prediction based on the combined consideration of surface characteristics and contact mechanics can support the design of sealing and mating surfaces and improve joint tightness, thereby helping to limit fluid losses and fugitive emissions.

## Figures and Tables

**Table 1:** Selected experimental studies on the effect of surface characteristics on sealing performance in joints with metal gaskets.

Type and Material of the Gasket and Sealed Surfaces	Roughness Parameter and Value	Surface Manufacturing Method	Load or Contact Pressure	Test Fluid	Measured Leakage Rate	Ref.
Gasket: flat copper gasket Sealed surfaces: unalloyed steel flanges	Parameter: maximum height of the roughness profile, without clear indication of the standard parameter Gasket surface: 1.5 µm Sealed surfaces: 11.5 µm	Gasket surface: polished Sealed surfaces: turned	Contact pressure: 0–120 MPa	Nitrogen at a pressure of 882 kPa	from ≤10^−5^ to 10^0^ L/h	[67]
Gasket: corrugated gasket manufactured from SUS304 steel Sealed surfaces: steel flanges	Parameter: Ra Gasket surface: no data Sealed surfaces: Ra = 1.5, 2.5, and 3.5 µm	No data	Axial force: 40–120 kN	Helium	from ≤10^−6^ to 2 × 10^−4^ Pa·m^3^/s	[60]
Gasket: flat copper gasket Sealed surfaces: 1045 steel joint	Parameter: Ra Gasket surface: Ra = 0.8 µm Sealed surfaces: Ra = 4 µm	Gasket surface: no data Sealed surfaces: turned	Axial force: 0.45–10.61 kN	5W-30 oil	from 10^−7^ to 10^−1^ mL/s	[34]
Gasket: flat gasket manufactured from SKD11 and 316 L steel Sealed surfaces: silicon wafer	Parameter: Ra Gasket surface: Ra = 0.3, 2.5, and 3.7 µm Sealed surfaces: no data	Gasket surface: forged, turned, and electrical discharge machined Sealed surfaces: no data	Axial force: 100–500 N	Water at a pressure of 0–0.3 MPa	from 1.4 × 10^−10^ to 2.3 × 10^−9^ m^3^/s	[13]
Gasket: three-layer corrugated gasket; outer layers manufactured from C1020 oxygen-free copper, inner layer manufactured from SUS304 steel Sealed surfaces: SS400 steel flanges	Parameter: Ra Gasket surface: no data Sealed surfaces: Ra = 2.5 and 3.5 µm	No data	Axial force: 40–120 kN	Helium	from 9 × 10^−10^ to 5 × 10^−7^ Pa·m^3^/s	[66]
Gasket: sharp-edged gasket manufactured from Alloy 800HT Sealed surfaces: clamping plates manufactured from Inconel Alloy 625	Parameter: Ra Gasket surface: no data Sealed surfaces: Ra = 4.2 µm	No data	Contact pressure: 50–600 MPa	Helium at a pressure of 40 bar; tests conducted at 20, 200, and 400 °C	from 10^−7^ to 10^0^ mg/(m·s)	[61]

**Table 2:** Selected rough-surface contact models.

Type of Contact	Formula for the Real Area of Contact	Formula for the Load, Where Applicable	Model Authors	Ref.
Elastic	$A_{r}=\pi r_{0}^{2}=\pi Rs$ where: $r_{0}$—radius of the circular contact area R—sphere radius s—indentation	$F=\frac{4}{3}E^{*}s^{\frac{3}{2}}R^{\frac{1}{2}}$ where: $E^{*}$—equivalent elastic modulus s—indentation R—sphere radius	Hertz	[71]
Elastic	$A_{r}\propto F^{n},n\to 1$ where: F—load	Not applicable	Archard	[73]
Elastic	$A_{r}=\pi \eta A_{n}R\sigma F_{1}\left(h\right)$ where: η—asperity density $A_{n}$—nominal contact area R—asperity radius of curvature σ—standard deviation of the asperity height distribution $F_{1}\left(h\right)$—auxiliary function for the contact area	$F=\frac{4}{3}\eta A_{n}E^{*}R^{\frac{1}{2}}\sigma^{\frac{3}{2}}F_{\frac{3}{2}}\left(h\right)$ where: η—asperity density $A_{n}$—nominal contact area $E^{*}$—equivalent elastic modulus R—asperity radius of curvature σ—standard deviation of the asperity height distribution $F_{\frac{3}{2}}\left(h\right)$—auxiliary function for the load	Greenwood, Williamson	[79]
Elastic-plastic	$A_{r}\left(d\right)$$=\eta A_{n}\pi R\left[\int_{d}^{d+\omega_{c}}\left(z-d\right)\phi \left(z\right)dz+\right.$ $\left.+\int_{d+\omega_{c}}^{\infty }[2\left(z-d\right)-\omega_{c}]\phi \left(z\right)dz\right]$ where: η—asperity density $A_{n}$—nominal contact area R—asperity radius of curvature d—separation $\omega_{c}$—critical interference z—asperity height ϕ—asperity height distribution function	$F\left(d\right)=\eta A_{n}E^{*}\{\frac{4}{3}R^{\frac{1}{2}}\int_{d}^{d+\omega_{c}}{\left(z-d\right)}^{\frac{3}{2}}\phi \left(z\right)dz+$ $+\pi RK\frac{H}{E^{*}}\int_{d+\omega_{c}}^{\infty }[2\left(z-d\right)-\omega_{c}]\phi \left(z\right)dz\}$ where: η—asperity density $A_{n}$—nominal contact area $E^{*}$—equivalent elastic modulus R—asperity radius of curvature d—separation $\omega_{c}$—critical interference z—asperity height ϕ—asperity height distribution function K—maximum contact pressure factor H—hardness of the material	Chang, Etsion, Bogy	[82]
Elastic-plastic	$A_{r}=\frac{D}{2-D}a_{l}$ where: D—fractal dimension of the surface profile $a_{l}$—largest contact spot area	Elastic-plastic load (for D≠1.5): F $=A_{n}E^{*}\frac{4\sqrt{\pi }}{3}{G^{*}}^{\left(D-1\right)}g_{1}\left(D\right){A_{r}^{*}}^{\frac{D}{2}}[{\left(\frac{\left(2-D\right)A_{r}^{*}}{D}\right)}^{\frac{3-2D}{2}}+$ $-{a_{c}^{*}}^{\frac{3-2D}{2}}]+K\phi g_{2}(D){A_{r}^{*}}^{\frac{D}{2}}{a_{c}^{*}}^{\frac{2-D}{2}}$ Elastic-plastic load (for D=1.5): $F=A_{n}E^{*}\sqrt{\pi }{G^{*}}^{\frac{1}{2}}{\left(\frac{A_{r}^{*}}{3}\right)}^{\frac{3}{4}}\ln\left[\frac{A_{r}^{*}}{3a_{c}^{*}}\right]+\frac{3K\phi }{4}{\left(\frac{A_{r}^{*}}{3}\right)}^{\frac{3}{4}}{a_{c}^{*}}^{\frac{1}{4}}$ Plastic load: $F=A_{n}E^{*}K\phi A_{r}^{*}$ where: $A_{n}$—nominal contact area $E^{*}$—equivalent elastic modulus $G^{*}$—dimensionless fractal roughness parameter D—fractal dimension of the surface profile $g_{1}\left(D\right)$—function of the fractal dimension D $A_{r}^{*}$—dimensionless real contact area $a_{c}^{*}$—dimensionless critical contact spot area K—hardness coefficient ϕ—material property $g_{2}(D)$—function of the fractal dimension D	Majumdar, Bhushan	[74]
Elastic-plastic	$A_{r}=\frac{D}{2-D}a_{l}$ where: D—fractal dimension of the surface profile $a_{l}$—largest contact spot area	Total plastic load: $F_{p}=HA_{rp}$ Elastic load for $n_{min}+1<n<n_{ec}$: $F_{ee}$ $=\sum_{n=n_{min}}^{n=n_{ec}}\frac{4\pi^{\frac{1}{2}}E^{*}G^{D-1}Da_{l}^{\frac{D}{2}}}{3\left(3-D\right)l^{D}}{\left(\frac{\pi }{g}\right)}^{\frac{3-D}{2}}\left(1-\frac{1}{\gamma^{3-D}}\right)l^{3-2D}$ Elastic load for levels between $n_{pc}$ and $n_{ec}$: $F_{epe}$ $=\sum_{n=n_{ec}}^{n=n_{pc}}\frac{4\pi^{\frac{1}{2}}E^{*}G^{D-1}Da_{l}^{\frac{D}{2}}}{3\left(3-D\right)l^{D}}\left({\left(\frac{1}{\pi }{\left(\frac{\pi KH}{2E^{*}}\right)}^{2}\frac{l^{2D}}{G^{2D-2}}\right)}^{\frac{3-D}{2}}\right.+$ $\left.-{\left(\frac{\pi }{8\gamma^{2}}l^{2}\right)}^{\frac{3-D}{2}}\right)$ Elastic load due to the maximum scale: $F_{n_{min}}=\frac{4\pi^{\frac{1}{2}}E^{*}G^{D-1}Da_{l}^{\frac{D}{2}}}{3\left(3-D\right)l^{D}}\left(a_{l}^{\frac{3-D}{2}}-{\left(\frac{\pi }{8\gamma^{2}}l^{2}\right)}^{\frac{3-D}{2}}\right)$ Total elastic load: $F_{e}=F_{ee}+F_{epe}+F_{n_{min}}$ Total load: $F=F_{e}+F_{p}$ where: H—hardness of the material $A_{rp}$—real contact area in the plastic regime n—scale index $n_{min}$—minimum scale index considered $n_{ec}$—critical scale index for the transition from elastic to elastic-plastic contact $n_{pc}$—critical scale index for the transition from elastic-plastic contact to fully plastic contact $E^{*}$—equivalent elastic modulus G—fractal roughness parameter D—fractal dimension of the surface profile $a_{l}$—largest contact area l—length scale K—hardness coefficient γ—frequency spectrum of the surface roughness	Miao, Huang	[85]
Elastic-plastic	$A_{r}=\frac{D}{2-D}\varphi^{\frac{2-D}{2}}a_{l}$ where: D—fractal dimension of the surface profile φ—domain extension factor $a_{l}$—largest contact spot area	Load for elastic deformation: $F_{re}=\frac{D}{3-D}\phi^{\frac{2-D}{2}}\frac{4E^{*}\pi^{\frac{1}{2}}G^{D-1}}{3l^{D}}a_{ec}^{\frac{3-D}{2}}a_{l}^{\frac{D}{2}}$ Load for first elastic-plastic deformation: $F_{rep1}=\frac{D}{3}KH\phi^{\frac{2-D}{2}}\frac{1.2816a_{ec}^{-0.2544}}{1.425-0.568D}\left[a_{epc}^{1.2544-0.5D}+\right.$ $\left.-a_{ec}^{1.2544-0.5D}\right]a_{l}^{\frac{D}{2}}$ Load for second elastic-plastic deformation: $F_{rep2}=\frac{D}{3}KH\phi^{\frac{2-D}{2}}\frac{1.7176a_{ec}^{-0.1021}}{1.263-0.573D}\left[a_{pc}^{1.1021-0.5D}+\right.$ $\left.-a_{epc}^{1.1021-0.5D}\right]a_{l}^{\frac{D}{2}}$ Load for fully plastic deformation: $F_{rp}=\frac{HD}{2-D}\phi^{\frac{2-D}{2}}\left[a_{l}^{\frac{2-D}{2}}-a_{pc}^{\frac{2-D}{2}}\right]a_{l}^{\frac{D}{2}}$ Total load: $F=F_{re}+F_{rep1}+F_{rep2}+F_{rp}$ where: D—fractal dimension of the surface profile ϕ—domain extension factor $E^{*}$—equivalent elastic modulus G—fractal roughness parameter $a_{l}$—largest contact spot area $a_{ec}$—critical elastic contact spot area $a_{epc}$—first critical elastic-plastic contact spot area $a_{pc}$—critical plastic contact spot area l—length scale K—hardness coefficient H—hardness of the material	Yuan, Gan, Liu, Yang	[86]

**Table 3:** Selected leakage models at metal-to-metal interfaces.

Leakage Rate Equation	Flow Regime	Fluid Type (Model Applicability)	Model Authors	Ref.
$Q_{v}=\frac{L_{y}}{L_{x}}\sigma_{eff}\Delta P$ where: $L_{y}$—length of the nominal contact region transverse to the flow direction $L_{x}$—length of the nominal contact region in the flow direction $\sigma_{eff}$—effective flow conductivity of the gap ΔP—pressure drop	Laminar, viscous	Incompressible	Persson	[103]
$Q_{v}=C_{1}\frac{P_{1}-P_{2}}{9\pi \eta b}\frac{D}{7-4D}{\left(\frac{2-D}{D}\right)}^{\frac{7-3D}{2}}\psi^{\frac{\left(2-D\right)\left(3D-5\right)}{2}}$ ${\left(g_{1}\left(D\right)C\right)}^{\frac{3}{2}}{\left(A_{n}-A_{r}\right)}^{\frac{7-3D}{2}}e^{\left(-C_{2}S_{G}\right)}$ where: $C_{1}$—regression coefficient $P_{1}$—pressure on the high-pressure side $P_{2}$—pressure on the low-pressure side η—dynamic viscosity of the fluid b—gasket width D—fractal dimension of the surface profile ψ—expansion coefficient of the size distribution range $g_{1}\left(D\right)$—function of the fractal dimension D C—scale coefficient $A_{n}$—nominal gasket area $A_{r}$—real contact area $C_{2}$—regression coefficient $S_{G}$—average compressive stress of the gasket	Laminar, viscous	Incompressible	Feng, Gu, Zhang	[104]
$Q_{v}=\frac{2\pi K_{v}h\left(P_{0}-P_{t}\right)}{\eta \ln\frac{r_{t}}{r_{0}}}$ where: $K_{v}$—permeability h—height of the percolation channel $P_{0}$—internal pressure $P_{t}$—external pressure η—dynamic viscosity of the fluid $r_{0}$—inner radius of the gasket $r_{t}$—outer radius of the gasket Permeability: $K_{v}=\frac{\pi D_{f}\lambda_{max}^{4}}{128\tau (4-D_{f})S}$ where: $D_{f}$—fractal dimension of tortuous capillaries $\lambda_{max}$—maximum capillary diameter τ—tortuosity of the flow channels S—flow cross-sectional area	Laminar, viscous	Incompressible	Zhang, Chen, Huang, Chen	[12]
$Q_{v}=\frac{\left(\Delta P_{h}+\Delta P_{c}+\Delta P_{v}\right)h^{2}}{12\eta l}S$ where: $\Delta P_{h}$—pressure drop caused by hydrostatic pressure $\Delta P_{c}$—pressure drop caused by microcapillarity $\Delta P_{v}$—viscous pressure drop h—gap height S—cross-sectional area of oil in the y–z direction η—dynamic viscosity of the fluid l—oil leakage path length in the x direction	Laminar, viscous	Incompressible	Gu, Dai, Ma, Huang, Wang	[114]
$Q_{v}=\frac{P_{2}-P_{1}}{\sum_{i=1}^{n}\left[RF\left(i\right)\cdot \delta r\right]}$ where: $P_{2}$—fluid pressure on the high-pressure side $P_{1}$—pressure on the low-pressure side i—number of the successive element/node n—total number of elements/nodes RF(i)—local flow resistance of the i-th element/node δr—element/node length	Laminar, viscous	Incompressible	Zhang, Wang, Huang, Xu, Zhou	[115]

## Data Availability

No new data were created or analyzed in this study. Data sharing is not applicable to this article.

## Abbreviations

The following abbreviations are used in this manuscript:

AI	Artificial intelligence
CFD	Computational fluid dynamics
DT	Digital twin
FEM	Finite element method
ML	Machine learning
